# Global StationaryOT: trajectory inference for aging time courses of single-cell snapshots

**DOI:** 10.1093/bioinformatics/btag532

**Published:** 2026-07-23

**Authors:** Cole Boyle, Elias Ventre, Geoffrey Schiebinger

**Affiliations:** Department of Mathematics, University of British Columbia, Vancouver, BC V6T 1Z2, Canada; COMPutational Pharmacology and Clinical Oncology, CNRS, INSERM, CRCM, Centre Inria d’Université Côte d’Azur, Aix Marseille Univ, Institut Paoli-Calmettes, Marseille, 13005, France; Department of Mathematics, University of British Columbia, Vancouver, BC V6T 1Z2, Canada; Premium Research Institute for Human Metaverse Medicine (PRIMe), University of Osaka, Suita, Osaka 565-0871, Japan

## Abstract

**Motivation:**

Trajectory inference (TI) methods for single-cell snapshots of developmental systems have yielded numerous insights into the gene regulatory networks (GRNs) that control cell differentiation. Many TI algorithms have been proposed for recovering cell trajectories from single samples containing cells spanning a spectrum of differentiation states; however, these methods cannot leverage temporal information when a time course of such diverse samples is available. As interest grows in understanding how the regulation of GRNs changes as an organism ages, current TI theory and methods must be adapted to take advantage of all information in aging time courses of single-cell data.

**Results:**

In this paper, we present our novel age-conscious method, global StationaryOT, which exploits the temporal information in aging time courses to simultaneously reconstruct debiased cell trajectories at all ages. We demonstrate that this first-of-its-kind method achieves more accurate, biologically consistent trajectories in synthetic and real biological contexts where data sparsity produces significant noise in the outputs of current TI methods when they are applied to time course samples independently.

**Availability:**

An open-source Python implementation of global StationaryOT, including documentation and examples, is available at https://github.com/ColeBoyle/global-stationaryOT. The source code, data processing scripts, and processed data for reproducing the results in this paper are archived at https://doi.org/10.5281/zenodo.20723235. The raw hematopoiesis data from Li et al. (The dynamics of hematopoiesis over the human lifespan. *Nat Methods* 2025;22 422–34.) can be accessed at https://www.ncbi.nlm.nih.gov/geo/query/acc.cgi? acc=GSE189161.

## 1 Introduction

**Figure 1:**
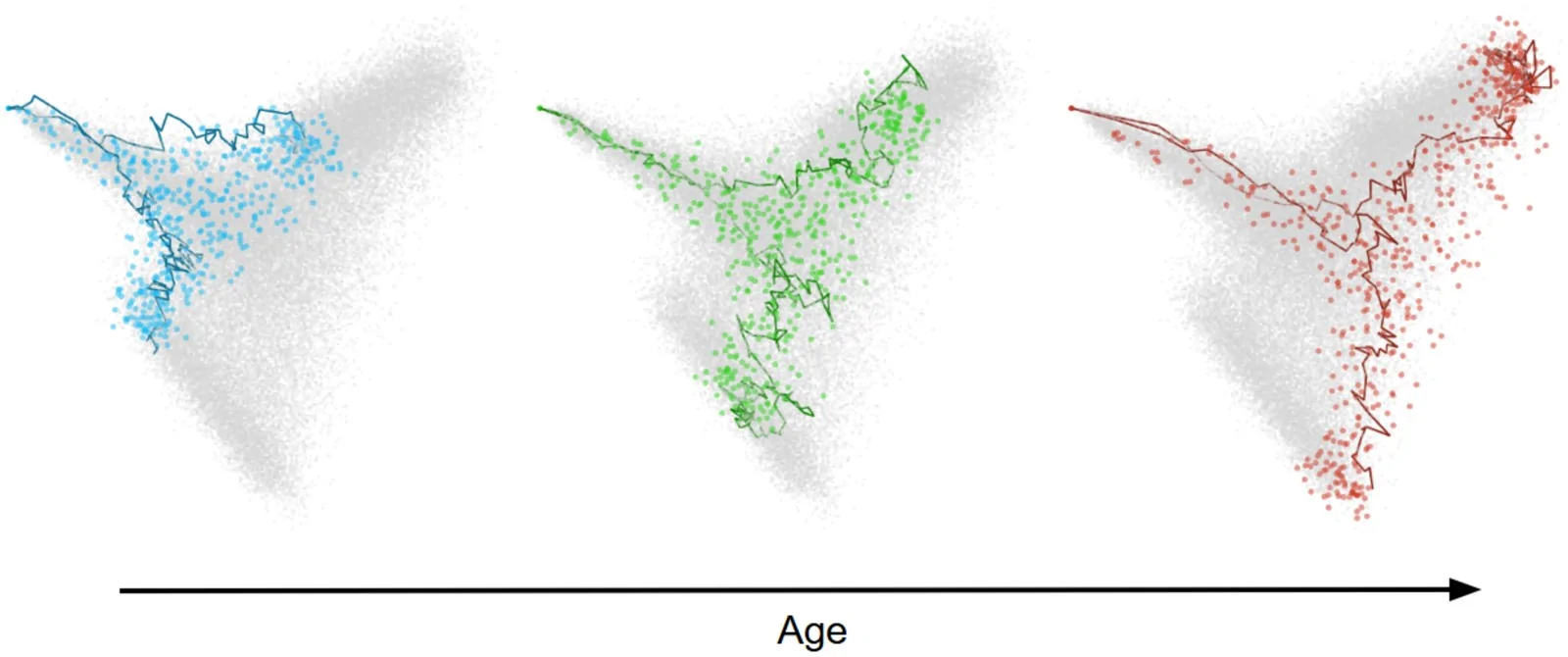
Simulated two-lineage aging single-cell time course. As the system ages, the lineages extend and rotate in the gene expression space. Sampled data at initial, intermediate, and final ages are coloured, along with sample cell trajectories, while data sampled at other ages are shown in grey.

Trajectory inference (TI) is a staple method for predicting the genes driving cell differentiation from single-cell data sets. Numerous methods have been proposed across a wide array of application domains (Trapnell *et al.* 2014, Street *et al.* 2018, Weinreb *et al.* 2018, Bergen *et al.* 2020, Zhang *et al.* 2021, Weiler *et al.* 2024), many of which have led to new discoveries by identifying or filling in the gene regulatory networks (GRNs) responsible for driving cells towards committed types. However, since these methods operate on data sampled around a single point in time, questions often remain as to the manner in which the regulation of these GRNs changes as an organism ages. To answer these questions, experimentalists have begun producing time courses of developing cells from organisms at various ages (Li *et al.* 2025); see Fig. 1 for a simulated example. TI analyses of these time courses have thus far treated each sample independently, running the risk of over-fitting sampling noise that can degrade age-related signals. In this work, we propose a novel TI method specifically designed to reconstruct cell trajectories at all ages in these time courses simultaneously. Our method shares information globally across all ages, smoothing disparities in the relative abundance of cell types caused by sampling effects and allowing for more consistent trajectory estimates and the recovery of clearer aging signals.

At a given organism age, the persistent and stable cycle of cell growth, differentiation, and death allows many developmental processes to be viewed as near stationary; i.e. the population of cells across differentiation states is in equilibrium over short time scales. With this assumption, we model the developmental system at all ages as quasi-static (Douce 2011, Hoppenau and Engel 2013). We reason that under normal homeostatic conditions, organisms rely on having steady populations of differentiated cells, so any age-related changes cannot occur quickly enough to significantly disrupt the balance of cell states at any age. This quasi-static assumption underlies our method’s approach to reconstruction and regularization.

At any age, we can recover cell trajectories via the mass displacement necessary to restore equilibrium after a short period of growth (Weinreb *et al.* 2018, Zhang *et al.* 2021). We can then debias this reconstruction process using temporal information by (i) imposing that the system evolve slowly enough such that the cell distribution does not change rapidly between consecutive ages and (ii) strictly enforcing the equilibrium condition at each age. The first form of regularization ensures a smooth transition between the reconstructions, reducing overfitting due to general sampling noise. The second form is critical in cases where progenitor and terminal cell states are missing from our samples at certain ages. The imposition of equilibrium can force the inclusion of these states in the reconstruction when they are absent from a particular age, but present in nearby samples. Our method, global StationaryOT, balances these regularization principles with a data-fitting term to provide debiased trajectory reconstructions at all ages across a time course.

*Related work:* The first trajectory inference techniques were developed for single-cell snapshots of a developmental system sampled at a single age (Trapnell *et al.* 2014, Street *et al.* 2018). Since cells in these snapshots are unordered, a critical goal for these methods was the (mostly) unsupervised inference of a so-called pseudotime that sorts cells by their state of differentiation. Later methods enabled improved ordering estimates by utilizing additional information, such as RNA velocity (Bergen *et al.* 2020) or cell growth rates (Zhang *et al.* 2021). Modelling single snapshots as coming from a stationary system was originally proposed by Weinreb *et al.* (2018) and used to reconstruct cell paths via their method Population Balance Analysis (PBA). This assumption was later used by Zhang *et al.* (2021) to build their optimal transport-based method StationaryOT, on which our method is based.

After the original TI methods were proposed for snapshot data, it was realized that inference could be further improved by sampling cells as they develop in real-time (Schiebinger *et al.* 2019, Tong *et al.* 2020). Such real-time time courses are formed by periodically sampling a population of developing cells so that the samples are already approximately sorted by differentiation state, allowing TI to proceed by linking cells taken at consecutive times (Schiebinger *et al.* 2019). In contrast, we consider time courses of snapshots that ideally consist of cells across a spectrum of differentiation states, such that without regularization, cell trajectories are confined to the states present in a single sample. The setting we deal with can be viewed as an abstraction of the one considered when developing previous time course-based methods, as we have access to time-stamped cells, but these time stamps are no longer (necessarily) correlated with the development stage of the cells. In particular, our method extends the regularization ideas developed for real-time time courses, particularly those of Lavenant *et al.* (2024) and their Wasserstein regression scheme, to the setting of quasi-static aging. An illustration of the differences between these settings and methods is given in Fig. S1.1.

## 2 Methods

### 2.1 Quasi-static modelling of aging cell development

Let $X\subset R^{G}$ be the space of all possible gene expression states that cells can take across *G* genes. Each vector component represents the expression level of a particular gene. The effects of aging are modelled as a slowly evolving perturbation of the continuous developmental system, in which cell differentiation occurs significantly faster than the accrual of noticeable aging effects. As such, we introduce two time scales: the organism’s chronological age *t*, and its biological or physiological age a=ct, where *c* is the rate at which the organism ages. A specific cell at time *t* is modelled as a point, $X_{t}\in X$, with a trajectory described by the following stochastic equation


(1) $$dX_{t}=-\nabla \Psi (X_{t},a)dt+\sigma dW_{t}$$

where Ψ is an unknown epigenetic landscape. We have expanded upon previous models (Weinreb *et al.* 2018, Zhang *et al.* 2021) by allowing Ψ to evolve slowly with age. This addition is consistent with the observed correlation between structural epigenetic changes and an organism’s age (López-Otín *et al.* 2013, Benayoun *et al.* 2019, Lu *et al.* 2023, Wu *et al.* 2024, Amaral *et al.* 2026). $\sigma dW_{t}$ is an increment of standard Brownian motion with diffusivity $\sigma^{2}$, modelling the unaccounted for perturbations impacting the cell’s velocity, e.g. thermal fluctuations influencing the rate of DNA transcription. Cells also divide and exit the system with age-dependent exponential rates $\alpha (X_{t},a)$ and $\beta (X_{t},a)$, respectively, i.e. over an infinitesimal interval, *dt*, they have a probability $\alpha (X_{t},a)dt$ of dividing and probability $\beta (X_{t},a)dt$ of dying or leaving the system.

Given this model for individual cell trajectories with birth and death, the population distribution, ρ(x,t), over X of all cells at age *a* satisfies the following population balance PDE: $\partial_{t}\rho (x,t)=L\rho (x,t)$, with L given by


(2) $$\begin{array}{cc} L\rho (x,t)=\nabla \cdot (\nabla \Psi (x,a)\rho (x,t))+\frac{\sigma^{2}}{2}\nabla^{2}\rho (x,t) & \\ +R(x,a)\rho (x,t) & \end{array}$$

where R(x,a)=α(x,a)−β(x,a) is the age-dependent growth rate of cells at the gene-expression state x∈X. States with R(x,a)>0 are sources, where cells are actively proliferating, while states with R(x,a)<0 are sinks, as cells may die or leave the system in such locations.

Now, suppose the organism has reached a biological age *a* and has populations of cells spanning all levels of differentiation. Let the average lifespan of cells born at this age be denoted by ℓ(a). In developmental systems for which there is a continuous cycle of cell division, differentiation, and death, if the aging rate, *c*, is small enough, the system will remain in approximate equilibrium over the course of a single cell life cycle. More precisely, over a time interval, Δτ=O(ℓ(a)), if ℓ(a)≪1, then Δa=cΔτ≪1. So *a*, and hence the system, will have undergone a negligible change over Δτ and yet individual cells will, on average, have undergone a complete life cycle. Thus, over the interval (t,t+Δτ), the normalized population distribution $\bar{\rho }:=\rho /\int_{X}\rho$ will remain close to a steady-state distribution, $\tilde{\rho }(x;a):={\tilde{\rho }}_{a}$, obtained from solving


(3) $$L_{a}\tilde{\rho }=0,\;\int_{X}\tilde{\rho }=1,$$

where $L_{a}$ is obtained from L by treating *a* as a fixed parameter. In the thermodynamics literature, such a system is considered to be quasi-static (Douce 2011, Hoppenau and Engel 2013), or “slow” (Oono and Paniconi 1998), particularly in the limit when the process (here aging) occurs infinitely slowly and the system is precisely described by an infinite sequence of steady state distributions, such as those solving (3). Since it is critical to our trajectory recovery method, we state this quasi-static assumption (QSA) explicitly as


(QSA) $$\begin{array}{c} \text{At}\;a\;\text{given}\;\text{biological}\;\text{age}\;a,\;\text{for}\;t\in (a/c,a/c+\Delta \tau ),\\ \;\bar{\rho }(x,t)\approx \tilde{\rho }(x;a)\\ \;\text{where}\;\Delta \tau =O(\ell (a))\;\text{and}\;\tilde{\rho }(x;a)\;\text{satisfies}\;(3). \end{array}$$

We note that the quasi-static assumption does not apply to systems that have been sampled during periods of rapid change or to those experiencing a sudden shock, e.g. due to sickness or serious injury.

Having described the system’s behaviour over short periods of time, we move to a characterization of the aging dynamics. Letting ${\bar{\rho }}_{a}:=\bar{\rho }(\cdot ,a/c)$, the curve defined by the function $a\mapsto {\bar{\rho }}_{a}$ continuously conserves mass and hence will satisfy a continuity equation


(4) $$\partial_{a}{\bar{\rho }}_{a}+\nabla \cdot (\nabla \Phi_{a}{\bar{\rho }}_{a})=0,$$

where $\Phi_{a}(x):=\Phi (x,a)$ is a potential guiding the aging quasi-steady-state distribution. Note that, unlike Ψ, the potential Φ is not steering the differentiation of individual cells, but rather the entire distribution of developing cells, including fluctuations in cell densities due to division and death. Now, by identifying the density ${\bar{\rho }}_{a}$ with its corresponding probability distribution on X, $a\mapsto {\bar{\rho }}_{a}$ can be viewed as an absolutely continuous curve in the Wasserstein-2 space of probability distributions, $W_{2}(X)$ (Ambrosio *et al.* 2005). Thus, when Δa is sufficiently small, ${\bar{\rho }}_{a}$ and ${\bar{\rho }}_{a\pm \Delta a}$ should be close with respect to the Wasserstein-2 distance, $W_{2}$, and the movement of the distribution from ${\bar{\rho }}_{a}$ to ${\bar{\rho }}_{a+\Delta a}$ can be approximated using the optimal coupling.

#### 2.1.1 Observation model

Given the above model, experimental observations of the system at an organism’s chronological age *t*, or equivalently, the biological age a=ct, are finite samples drawn from the marginal distribution ${\bar{\rho }}_{a}$ by sequencing cells. That is, if we measure the state of $n_{a}$ cells $X_{1}^{a},\ldots ,X_{n_{a}}^{a}$ at age *a* then $X_{j}^{a}∼{\bar{\rho }}_{a}$. We can represent this sample as an empirical distribution, ${\hat{\rho }}_{a}$ supported on ${\bar{X}}^{a}={\left\{X_{j}^{a}\right\}}_{j=1}^{n_{a}}$


(5) $${\hat{\rho }}_{a}=\frac{1}{n_{a}}\sum_{j=1}^{n_{a}}\delta_{X_{j}^{a}}.$$

where $\delta_{x}$ represents the Dirac point mass located at state x∈X. Repeating the sequencing experiment on the organism at multiple ages, $a_{1},\ldots ,a_{T}$, gives rise to a time series of such empirical distributions, ${\left\{{\hat{\rho }}_{a_{i}}\right\}}_{i=1}^{T}$.

In the following, we also assume knowledge of the cell growth rates $R_{ij}:=R(X_{j},a_{i})$, in the form of estimates denoted by ${\hat{R}}_{ij}\approx R_{ij}$. These growth rates can be estimated using cell cycle and apoptosis gene signatures to identify proliferating and dying cells (Schiebinger *et al.* 2019). Alternatively, prior biological knowledge of cell type specific growth can also be used once cell identities have been inferred with marker genes (Weinreb *et al.* 2018, Rahni and Birnbaum 2019, Zhang *et al.* 2021). While our method can exploit age-dependent growth rates, currently estimates are typically only available at the age the cell was measured, i.e. we will need to take ${\hat{R}}_{ij}=\hat{R}(X_{j})$ for all *i*. We adopt this approach in all our experiments below. Note that for brevity, we will often replace the subscript $a_{i}$ with *i*, e.g. ${\hat{\rho }}_{i}:={\hat{\rho }}_{a_{i}}$.

### 2.2 Methodology overview

Given a time course of single-cell samples, our goal is to recover estimates of the complete cell paths at every age we sample. Under the model proposed above, we will see below that this is possible provided we have estimates of the cell growth rates, $\hat{R}$, and noise level $\sigma^{2}$. Our method builds off previous equilibrium based trajectory inference techniques designed for single stationary samples (Weinreb *et al.* 2018, Zhang *et al.* 2021). Given cell growth rates, these methods recover cell trajectories as the necessary cell flow required to restore the population equilibrium after a short period of growth. Specifically, after cells divide, there will be more cell mass on high-growth regions, R(x,a)>0, than low-growth regions, R(x,a)<0. Absent cell movement, this growth would push the distribution out of equilibrium, or in our case, quasi-equilibrium. Hence, the cells’ flow can be recovered as the motion necessary to keep the population in a (quasi)-equilibrium. These methods fit a Markov transition matrix to the sampled data, ${\hat{\rho }}_{a}$, that approximates this flow and from which cell trajectories that trace paths from the high-growth to low-growth states can be sampled. Moreover, it can be used to compute the probability that any given cell in ${\hat{\rho }}_{a}$ ends up in a particular low-growth region, e.g. that it leaves the system as a particular terminal cell type.

**Figure 2:**
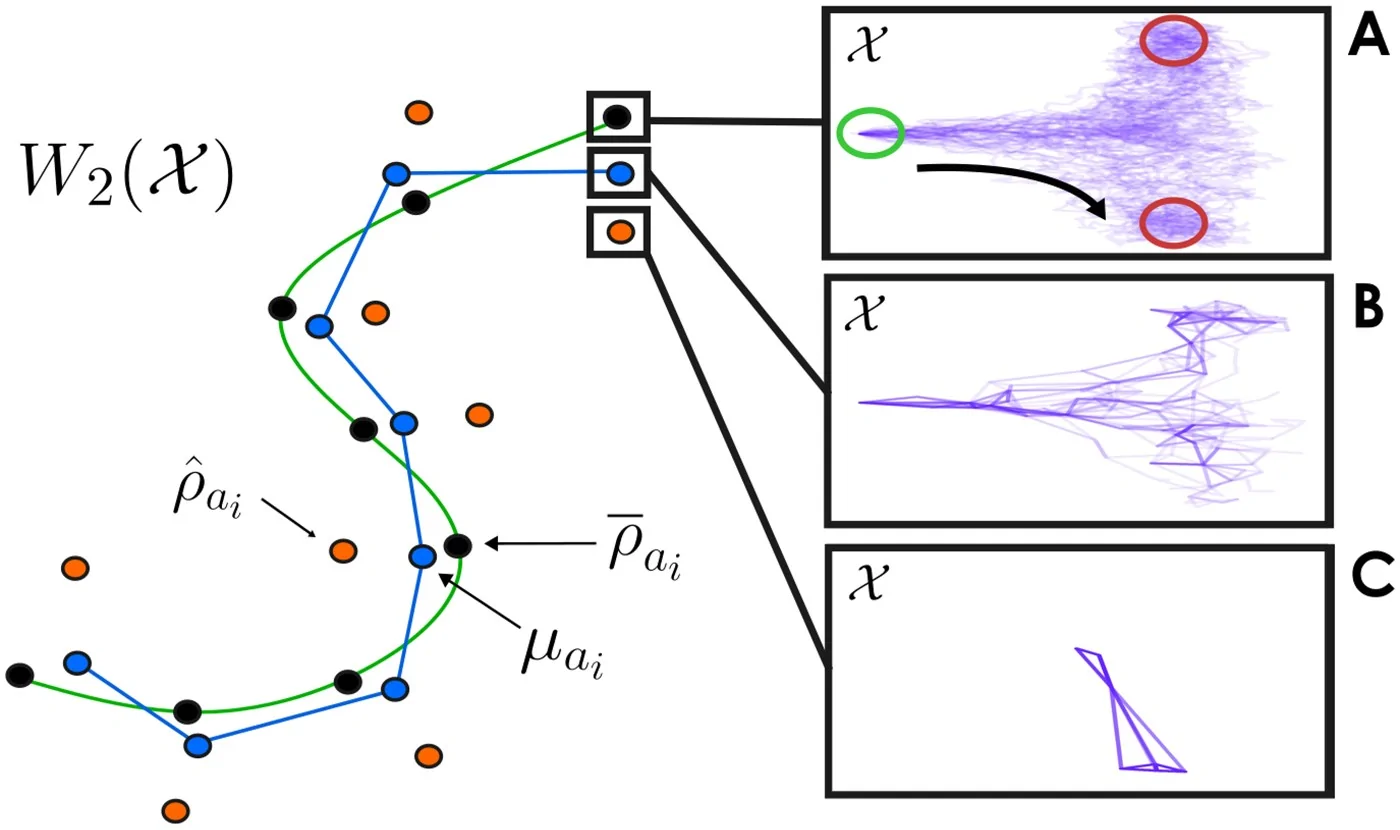
An abstract illustration of the quasi-static aging cell population curve, with sample and inferred cell paths displayed at the initial age. The green curve represents the aging cell population evolving according to (4) in the Wasserstein space, $W_{2}(X)$. At a particular age, $a_{i}$, the true normalized cell distribution is represented by a black dot. Orange dots represent our finite sample distributions, ${\hat{\rho }}_{a_{i}}$, taken by sequencing cells, $X∼{\bar{\rho }}_{a_{i}}$. Since they are finite, they can be quite far from the true distribution ${\bar{\rho }}_{a_{i}}$. Blue dots represent the cell distributions, $\mu_{a_{i}}$, reconstructed at each age using global StationaryOT (gStatOT). These reconstructions are connected to highlight gStatOT’s approach for improving inference by sharing information across samples to (i) smooth transitions in the population distribution between ages and (ii) balance cell growth at each age. We implement (i) by minimizing the smoothing term, in (14), which can be seen as attempting to bring the blue points closer together to better approximate the green curve with the blue curve, an approach analogous to the smoothing procedures used in standard regression problems to address overfitting. With (ii) we aim to use our QSA to infer more robust cell paths at each age using the chain of approximations $\mu_{a_{i}}\approx {\tilde{\rho }}_{a_{i}}\approx {\bar{\rho }}_{a_{i}}$, where ${\tilde{\rho }}_{a_{i}}$ is the solution to (3). (A) Shows the true cell trajectories at age $a_{1}$, traversed from primary sources (green region) to sink regions (red) in the gene space, X. gStatOT and StationaryOT (StatOT) trajectory reconstructions at the same age are shown in (B) and (C), respectively. The missing structure in (C) results from the single sample not containing enough information on its own (i.e. source and sink states) for StatOT to fully reconstruct both cell lineages starting from the primary source region.

Critically, these reconstruction procedures rely on the samples, ${\hat{\rho }}_{a}$, containing cells spread across the entire spectrum of differentiation states. When the ${\hat{\rho }}_{a}$ are sparse the inferred trajectories fail to correctly span this spectrum. In particular, missing cells in critical transition states will leave gaps in our trajectories, potentially causing spurious transitions. More importantly, when the samples fail to capture cells from every source and terminal region we may end up missing entire cell lineages (see the differences between Fig. 2A and C for an example)—if our sample contains cells of only a single type we cannot reconstruct the dynamics at all.

When a time course of samples is available our method generalizes the equilibrium approach to trajectory inference to the quasi-static setting and aims to alleviate overfitting sparsity by sharing all available information when fitting the transition matrices at each age. We accomplish this by formulating a constrained convex optimization problem over (unnormalized) transition matrices, $\pi_{a_{i}}$, that balances data fitting and regularization to fill gaps in the data as well as pull the inferred dynamics in line with those of the ground truth. Specifically, we aim to fit each $\pi_{a_{i}}$ to all available data so that it best approximates the pure equilibrium dynamics specified in (3), and hence by our QSA also approximates the true dynamics at each age specified by the population model in (2). In particular, we reconstruct an optimal equilibrium distribution, $\mu_{a_{i}}$, that better approximates ${\tilde{\rho }}_{a_{i}}$ than our raw data ${\hat{\rho }}_{a_{i}}$, while simultaneously fitting the cell dynamics, $\pi_{a_{i}}$, consistent with (3) and having solution $\mu_{a_{i}}$. Consequently, we use the chain of approximations $\mu_{a_{i}}\approx {\tilde{\rho }}_{a_{i}}\approx {\bar{\rho }}_{a_{i}}$ to build a curve $a\mapsto \mu_{a}$, with associated equilibrium cell dynamics, $\pi_{a}$, that approximate the true aging cell distribution curve $a\mapsto {\bar{\rho }}_{a}$ and the ground truth cell dynamics (2). See Fig. 2 for a conceptual visualization of these curves.

### 2.3 StationaryOT

Since our method extends StationaryOT (StatOT) (Zhang *et al.* 2021) to time series data, we briefly outline its methodology below. For a more detailed elaboration of the method, with benchmarks and the biological insights it has led to, see Li *et al.* (2025), Zhang *et al.* (2021), and Shahan *et al.* (2022).

Suppose that at age *a*, we know the stationary distribution ${\tilde{\rho }}_{a}$, solving $L_{a}\tilde{\rho }=0$. Although the population distribution remains unchanged, individual cells are still undergoing a cycle of division, differentiation, and death. Over a short interval Δt<ℓ(a), the StatOT method observes that these cell dynamics can be approximated by separating the effects of cell flux and transport, after which we can recognize that the resulting transport equation for cell movement can be solved using entropic optimal transport (EOT).

Specifically, $L_{a}$ is split into equations for growth and transport in the distributions ${\tilde{\rho }}_{G}$ and ${\tilde{\rho }}_{T}$ respectively. Setting $t_{0}=a/c$, these are given by,


(6) $$\begin{array}{c} \partial_{t}{\tilde{\rho }}_{G}(x,t;a)=R(x,a){\tilde{\rho }}_{G}(x,t)\\ {\tilde{\rho }}_{G}(x,t_{0};a)={\tilde{\rho }}_{a}(x). \end{array}$$

(7) $$\begin{array}{cc} \partial_{t}{\tilde{\rho }}_{T}(x,t;a)=\nabla \cdot (\nabla \Psi (x,a){\tilde{\rho }}_{T}(x,t;a)) & \\ +\frac{\sigma^{2}}{2}\nabla^{2}{\tilde{\rho }}_{T}(x,t;a) & \\ {\tilde{\rho }}_{T}(x,t_{0};a)={\tilde{\rho }}_{G}(x,t_{0}+\Delta t;a). & \end{array}$$

Notice that (6) applies the effects of cell growth to ${\tilde{\rho }}_{a}$, then, over this same period, Δt, (7) specifies the transport of this evolved distribution via a drift-diffusion process. The solution to (7), evaluated at $t_{0}+\Delta t$, is a *splitting approximation* of the equilibrium distribution ${\tilde{\rho }}_{a}(x)$. The two distributions coincide as Δt tends to 0, with a pointwise error of order $O({\left(\Delta t\right)}^{2})$ (Holden *et al.* 2010), i.e.


(8) $${\tilde{\rho }}_{a}(x)={\tilde{\rho }}_{T}(x,t_{0}+\Delta t)+O({\left(\Delta t\right)}^{2}).$$

Proceeding to solve these equations, by denoting $g(x;a,\Delta t)=e^{R(x,a)\Delta t}$, we can see that the solution to (6), evaluated at $t_{0}+\Delta t$, is given by ${\tilde{\rho }}_{G}(x,t_{0}+\Delta t;a)={\tilde{\rho }}_{a}(x)g(x;a,\Delta t)$.

With this solution, (7) and (8) specify that our cell dynamics over the period Δt are approximated by a drift-diffusion process with initial distribution ${\tilde{\rho }}_{G}(x,t_{0}+\Delta t;a)$ and final distribution ${\tilde{\rho }}_{a}$. Note that on short time scales, the diffusion ($O(\sqrt{\Delta t})$) dominates drift (O(Δt)) in (7). These diffusion-driven dynamics can be described by an EOT coupling, $\pi_{a}^{⋆}$, of ${\tilde{\rho }}_{G}$ and ${\tilde{\rho }}_{a}$. This is called a Schrödinger bridge; see Léonard (2012, 2014) for a review of this connection between diffusion and EOT. More precisely, the coupling $\pi_{a}^{⋆}$ estimating cell dynamics is the unique minimum of the convex optimization problem,


(StatOT) $${\text{OT}}_{\sigma^{2}\Delta t}({\tilde{\rho }}_{G},{\tilde{\rho }}_{a}):=\underset{\pi \in \Pi ({\tilde{\rho }}_{G},{\tilde{\rho }}_{a})}{\text{inf}}H_{\sigma^{2}\Delta t}(\pi )$$

where $\Pi (\gamma_{1},\gamma_{2})=\{\pi \in P(X\times X):P_{1\#}\pi =\gamma_{1},P_{2\#}\pi =\gamma_{2}\}$ is the set of all couplings between the distributions $\gamma_{1}$ and $\gamma_{2}$. Here, $P_{i}$ denotes the projection of a point $x\in R^{G}$ onto its $i^{\text{th}}$ coordinate and ${\;}_{\#}$ denotes the push-forward operator so that $P_{i\#}\pi =\int \pi (dx_{i},\cdot )$ is the $i^{\text{th}}$ marginal of π. Next,


(9) $$H_{\varepsilon }\left(\pi \right)=\int \int_{X\times X}\|x-y{\|}^{2}\pi \left(dx,dy\right)+\varepsilon H\left(\pi |\text{Leb}\right)$$

is the quadratic optimal transport loss with entropic regularization of strength ε. Leb is the Lebesgue measure on $X^{2}$ and $H(\gamma_{1}|\gamma_{2})=\int \;\text{log}\;(\frac{d\gamma_{1}}{d\gamma_{2}})d\gamma_{1}$ is the relative entropy between two distributions. To summarize the StatOT recovery process for a discrete sample of observations, ${\hat{\rho }}_{a}$, if we have growth rate estimates ${\hat{R}}_{ij}$ for the observed cells $X_{j}\in {\bar{X}}^{a}$ and an estimate of Δt and noise level $\sigma^{2}$ (see SI.2 for details on choosing these parameters), we first form the distribution ${\hat{\rho }}_{G}=\frac{1}{n_{a}}\sum_{j=1}^{n_{a}}{\hat{g}}_{ij}\delta_{X_{j}}$ where ${\hat{g}}_{ij}=e^{{\hat{R}}_{ij}\Delta t}$. We then couple ${\hat{\rho }}_{G}$ and ${\hat{\rho }}_{a}$ with entropic optimal transport using the regularization parameter $\varepsilon =\sigma^{2}\Delta t$ to obtain an estimate for the cell dynamics in the form of a coupling matrix $\pi_{a}^{⋆}$. After row-normalization, we can sample cell trajectories and compute cell-fate probabilities. See SI.3 for a description of how we compute these probabilities using the cell growth rates.

### 2.4 Global StationaryOT

As discussed in the overview, given measurements of the developmental process at a series of ages, ${\left\{{\hat{\rho }}_{a_{i}}\right\}}_{i=1}^{T}$, we propose a convex optimization problem that balances data fitting and regularization to reconstruct improved estimates of the $\pi_{i}^{⋆}$ by smoothing their supports, namely the equilibrium distribution $\mu_{i}:=P_{2\#}\pi_{i}^{⋆}$, across all the sampled ages. Letting $\bar{\pi }=(\pi_{1},\ldots ,\pi_{T})\in P{\left(X\times X\right)}^{T}$ be a vector of couplings abbreviating $g_{i}:=g(\cdot ;a_{i},\Delta t)$, our recovery program has the following form


(gStatOT) $$\underset{\bar{\pi }\in P{\left(X\times X\right)}^{T}}{\text{inf}}\lambda \mathrm{Reg}(\bar{\pi })+\mathrm{Fit}(\bar{\pi })$$

(10) $$\text{such}\;\text{that}\;P_{1\#}\pi_{i}=g_{i}\mu_{i},\forall i=1,\ldots ,T$$

where the **Fit** operator is an abstraction of the StatOT problem, allowing the second marginal of each $\pi_{i}$ to deviate from ${\hat{\rho }}_{i}$, while **Reg** encourages smooth transitions between the support of the $\pi_{i}$, consistent with a slow aging process.

#### 2.4.1 Constraint enforces equilibrium dynamics

Since the marginals of the $\pi_{i}$ can now move during optimization, the constraint ensures that the first marginal of $\pi_{i}$, $P_{1\#}\pi_{i}$, still represents an evolution of $\mu_{i}$ after a period of growth, i.e. we still enforce that $P_{1\#}\pi_{i}$ be a solution to (6) (evaluated at a/c+Δt). Moreover, together with the unity constraint, $\pi_{i}\in P(X\times X)$, (10) implies that our reconstructed $\mu_{i}$ will be equilibrium distributions for the given growth rates. Namely, each $\mu_{i}$ must specify balanced mass on source and sink regions such that the net cell flux is 0. Indeed, both constraints together imply that we must have $\int P_{1\#}\pi_{i}=\int g_{i}\mu_{i}=1$ and $\int \mu_{i}=1$. So splitting the domain as $X=X_{\text{source}}\cup X_{\text{sink}}\cup X_{0}$ for regions with $R_{i}>0,R_{i}<0$ and $R_{i}=0$, respectively, and noting that $g_{i}=1$ on $X_{0}$, we can see that


(11) $$\int_{X_{\text{source}}}g_{i}\mu_{i}+\int_{X_{\text{sink}}}g_{i}\mu_{i}+\int_{X_{0}}g_{i}\mu_{i}=1=\int_{X}\mu_{i}$$

(12) $$\Rightarrow \int_{X_{\text{source}}}\left(g_{i}-1\right)\mu_{i}=\int_{X_{\text{sink}}}\left(1-g_{i}\right)\mu_{i}.$$

The left-hand side is the mass gained after growth, while the right-hand side represents what is lost. We are thereby regularizing the $\mu_{i}$, and thus $\pi_{i}$, by strongly enforcing our equilibrium hypothesis that cells leaving the system are fully replaced at each age. As noted in the overview, this is critical, as equilibrium-based inference fails when a sample lacks source or sink states. In such cases, our $\mu_{i}$ will adapt and become equilibrium distributions by adding states from the missing regions in order to have $\int g_{i}\mu_{i}$ reach unity. In practice, these additional states can come from other samples in our time course. Through this sharing of state information we are also able to satisfy the natural solvability criterion that arises from (12) due to needing both sides of the equation to be nonzero (see SI.1.2 for a discussion of this criterion).

#### 2.4.2 Fitting functional optimizes dynamics at each age

We have constrained the $\pi_{i}$ to describe equilibrium dynamics without a priori knowledge of what their marginals should be. Now, we move to describing how we fit these $\pi_{i}$ to our time course so that they best approximate the dynamics described by (3), and hence by our QSA, the true cell dynamics implied by (2).

As laid out in the StationaryOT section, for a given estimate of the true equilibrium distribution (in that case ${\hat{\rho }}_{i}$ and in ours $\mu_{i}$) the cell dynamics consistent with (3) are obtained by minimizing the loss $H_{\sigma^{2}\Delta t}$ over couplings that, at least in theory, satisfy our constraint. To anchor these dynamics to each age, we keep each $\mu_{i}$ close to our sampled data ${\hat{\rho }}_{i}$, giving the data-fitting functional


(13) $$\mathrm{Fit}(\bar{\pi };w,\varepsilon_{2},0)=\sum_{i=1}^{T}\left[H_{\varepsilon_{2}}(\pi_{i})+w{\text{OT}}_{0}(\mu_{i},{\hat{\rho }}_{a_{i}})\right].$$

Here, each term in **Fit** extends the StatOT problem by penalizing the $\mu_{i}$ for deviating from ${\hat{\rho }}_{i}$ in squared $W_{2}$ distance, instead of enforcing them to be equal. The strength of this penalty term is controlled by the parameter *w* such that, when λ=0, taking w→∞ recovers the StatOT problem, with the additional constraint of imposing balanced growth. In practice, we have found that simply taking w=1 works well for sparse data, and so we set this as the default value for most of our experiments. Moreover, $\varepsilon_{2}$ plays the same role as ε in StatOT and is set according to the same principles. See SI.2 for details on choosing these parameters, along with the smoothing parameter λ.

#### 2.4.3 Regularization functional smooths transitions between ages

Now, following our observation that in the quasi-static limit, consecutive ${\bar{\rho }}_{a}$ should be close with respect to the $W_{2}$ metric, our QSA implies that the ${\tilde{\rho }}_{a}$ should be as well. Thus, to guide our reconstructed marginals, $\mu_{i}$, closer to ${\tilde{\rho }}_{a}$ and hence ${\bar{\rho }}_{a}$ (as depicted in Fig. 2) we define our **Reg** functional by the following


(14) $$\mathrm{Reg}(\bar{\pi };0)=\sum_{i=1}^{T-1}\frac{1}{\Delta a_{i}}{\text{OT}}_{0}(\mu_{i},\mu_{i+1}),$$

where $\Delta a_{i}=a_{i+1}-a_{i}$. While the evolution of the $\mu_{i}$ should happen on the aging timescale, since the rate *c* is generally unknown we absorb it into the regularization strength λ. So, in practice our regularization coefficient is given by $\frac{\lambda }{t_{i+1}-t_{i}}$, where $t_{i}$ is the chronological age at which we sampled ${\hat{\rho }}_{i}$. We emphasize that $t_{i+1}-t_{i}$ is assumed to be significantly larger than our previously defined cell state transition time step Δt.

#### 2.4.4 Implementation

To obtain a finite-dimensional convex optimization problem, we restrict gStatOT to measures supported on a finite set. Following Lavenant *et al.* (2024), we choose this set to be the union of all our sampled data, $\bar{X}$, since a standard gridding approximation is intractable due to the high dimensionality of X (see SI.1.1 for details). Upon discretization, the gStatOT problem is strictly convex over the couplings, $\pi_{i}$, and hence has a unique solution that we can solve for by maximizing the corresponding dual problem. We solve this dual problem using a block coordinate ascent (BCA) algorithm (Luo and Tseng 1992) with log-domain arithmetic, and use a tolerance on the duality gap as a stopping condition. See SI.1.3 and 1.4 for details on deriving the dual problem and our BCA implementation.

We provide Python implementations of gStatOT and StatOT using the JAX library (Blondel *et al.* 2022, Cuturi *et al.* 2022, Bradbury *et al.* 2025), enabling seamless execution on GPUs or CPUs. Both implementations are designed to run on single-cell data stored in a standard AnnData (Virshup *et al.* 2024) object and are available, along with detailed usage instructions, at https://github.com/ColeBoyle/global-stationaryOT.

## 3 Results

### 3.1 Synthetic data: aging epigenetic landscape

We demonstrate gStatOT’s ability to reconstruct trajectories in high-sampling-bias regimes on a simulated birth-death diffusion system representing the development and aging of two cell lineages in a 10-dimensional gene space. The differentiation of individual cells is driven by the dynamics in equation (1) with an age-dependent developmental landscape, i.e. Ψ(x,a). This landscape generates a bifurcating developmental pathway from a single high-growth region towards one of two sink regions (as shown in Fig. 2A). For clarity, we have centered the system so that the high-growth progenitors are located at the origin, and have chosen the unit of our chronological age, *t*, to be days. Cells are produced from this progenitor state and then develop by increasing their expression of Gene 1, followed by stochastically committing to one of two lineages by up- or down-regulating Gene 2, after which they eventually die. We refer to the two cell lineages as +2 and −2, respectively.

The developmental landscape’s dependence on age impacts the system by increasing the Gene 1 expression needed for commitment to occur, while also inducing an increase and corresponding decrease of Gene 3 in the +2 and −2 lineages, respectively. The precise mathematical details of our simulation can be found in SI.4.2. As in real data, only a minority of genes drive lineage development, while the rest add noise and dimensionality that confound inference. We simulated a time course with 25 ages by sampling all cells from the system every 4 days. Ground truth estimates of the cell trajectories and fate probabilities at each age were obtained by simulating trajectories starting from the sampled cell states at every age.

**Figure 3:**
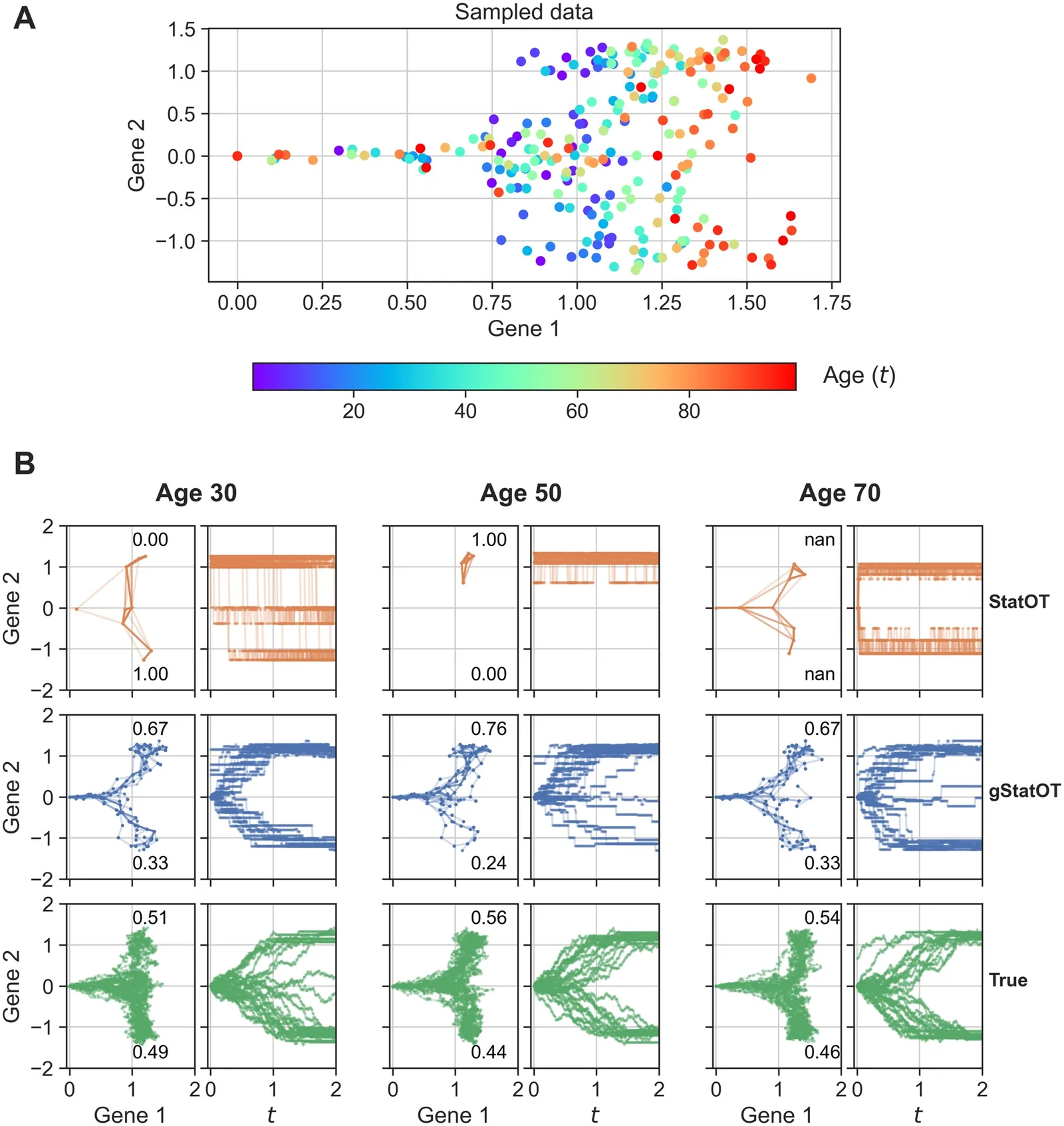
(A) Subsampled simulated data at 25 ages with 10 cells per age. (B) 25 2-day trajectories reconstructed from the top data using gStatOT and StatOT alongside 25 ground truth simulated trajectories at ages 30, 50, and 70. The adjacent plots show the (centered) Gene 2 expression of each trajectory as a function of its Gene 1 expression and chronological age, *t*, respectively. The numbers next to each lineage denote the proportion of trajectories that first reach either a +2 or −2 sink state. Note that there are no sink states in one, or both, of the lineages at each age. So while the StatOT trajectories can explore the state space at these ages, they are not able to reconstruct the full bifurcation structure of the system, and so, as with age 70, may fail to converge at all.

To introduce additional sampling bias, prior to applying our method we downsampled the time course to 10 cells per age. We applied gStatOT to the subsampled time course and compared the results to those obtained by applying StatOT to the same data at each individual age. See SI.4 for the details on how parameters were chosen and Fig. S1.2 for an analysis of gStatOT’s sensitivity to errors in growth rate estimates. Figure 3 shows the subsampled time course and 25 reconstructed trajectories from both gStatOT and StatOT alongside 25 of the simulated trajectories at several ages. We observe that the reconstructed gStatOT trajectories more closely match those of the ground truth. Moreover, the growth rate regularization motivates the inclusion of the source cells located at the origin in gStatOT’s marginals, allowing us to correctly reconstruct trajectories that start from the origin. In contrast, StatOT’s trajectories are forced to start from positive-growth cells in the +2 lineage at age 30.

Next, we sought to quantify gStatOT’s performance and how well it improves with increased sampling rates. We used three metrics to evaluate the performance of our method, and compared the results to those obtained with StatOT and PBA (Weinreb *et al.* 2018). With the first metric, we measure gStatOT’s ability to debias sparse samples by evaluating the $W_{2}$ distance between the reconstructed marginals ($\mu_{i}$) and all simulated data from each age. StatOT and PBA operate exclusively on the sparse samples at each age, so to test if the $\mu_{i}$ are actual improvements over the raw data, we also show the $W_{2}$ distance between these sparse samples and the full simulated data. The second metric compares the reconstructed trajectories to the ground truth cell paths we simulated at each age. As done in Zhang *et al.* (2021) and Lavenant *et al.* (2024), we choose to use the $W_{2}$ metric on path distributions. Our final performance metric compares the inferred cell-fate probabilities with those obtained from the simulation in total variation (TV) distance. When a method failed to reconstruct fate probabilities we set the TV value for that age to its maximum value of 1.

**Figure 4:**
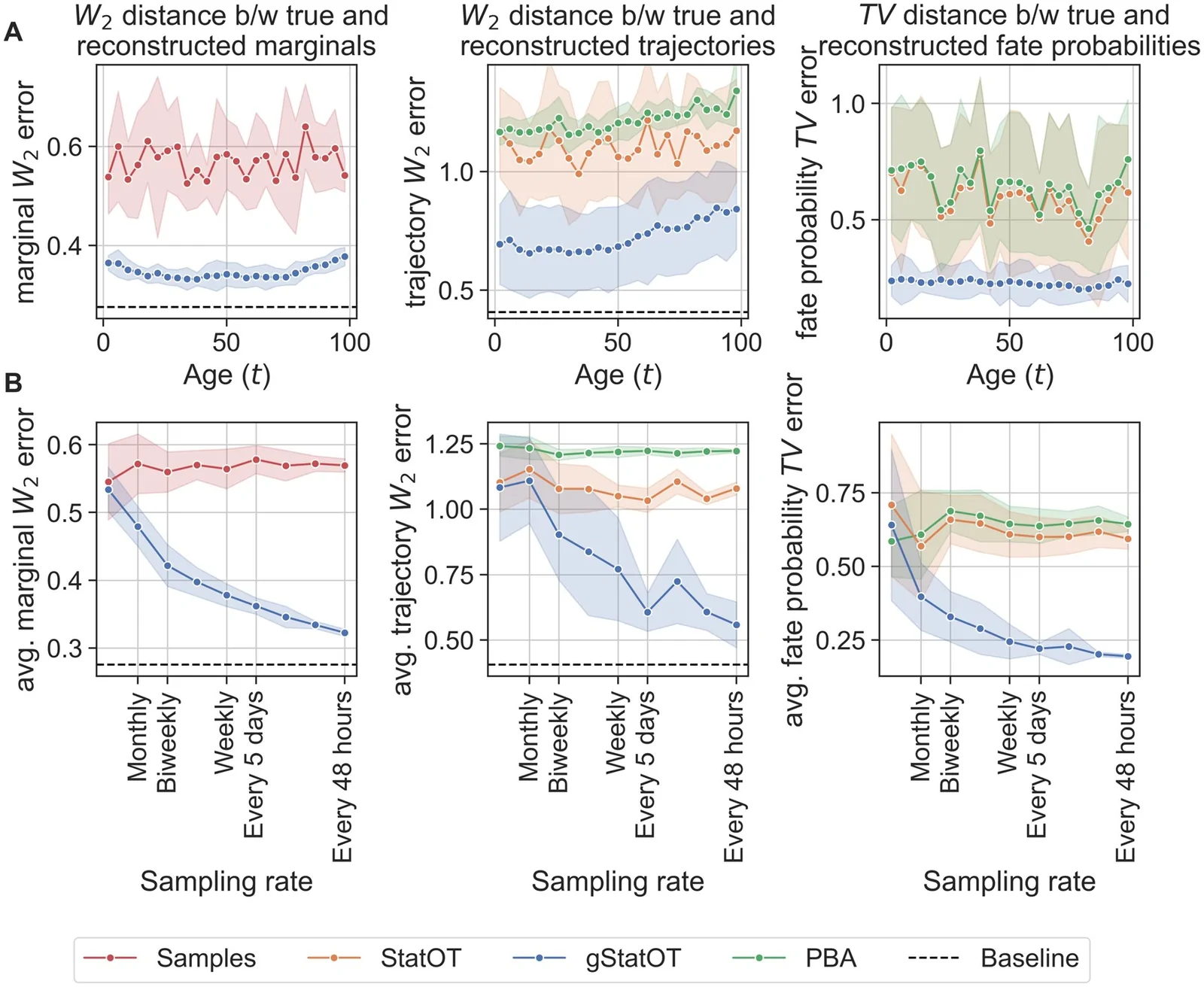
(A) Model performance across age in marginal, trajectory, and fate probability reconstruction for simulated data with 25 ages and $n_{a}=10$ cells per age. (B) Model performance for varying number of intermediate time points. The decreasing trends in all plots demonstrate that gStatOT is successfully transmitting information across ages to improve its estimates. The data for each sampling rate is the average of each metric across all ages. The results are summarized by the mean and standard deviation of each method’s performance on 10 independent reseeded simulations. The baseline represents the average distance between the original simulated distributions and those obtained from the 10 reseeded simulations.

Each model’s hyperparameters were chosen by optimizing the average of the three metrics on a single simulation (see SI.7.1). With these parameters, each method was then validated across 10 independent reseeded simulations, with the results being reported in Fig. 4. A baseline was established for the marginal and trajectory metrics by comparing the complete simulated data from the initial simulation to the 10 reseeded ones. Figure 4A shows that across all 25 sampled days in the time course, gStatOT was able to construct marginals closer to our ground truth simulated data than the subsamples and outperform StatOT and PBA in both the trajectory reconstruction and fate probability estimation. The results are summarized by the mean and standard deviation of each metric across the 10 independent simulations.

To test whether gStatOT is successfully exploiting information across multiple time points, we repeated the above process for time courses generated with variable sampling rates. Figure 4B shows the average performance of gStatOT, StatOT and PBA across all ages for each time course. We observe that gStatOT’s estimates increase in accuracy for each metric as the sampling frequency increases and reach near baseline once we are sampling every other day. On the other hand, those of StatOT and PBA remain relatively constant, providing evidence that gStatOT is successfully utilizing global information across the time course.

### 3.2 Synthetic data: loss of cellular identity over age induced by epigenetic erosion

Within several biological systems, a progressive loss of cell identity has been observed to occur over the course of the aging process (Traxler *et al.* 2023, Connolly *et al.* 2024, Amaral *et al.* 2026). Due to the effects this has on a cell’s ability to perform its specialized roles, it has been theorized that this loss of identity could play a critical role in bringing about the physiological changes and diseases generally associated with aging (Ono and Cutler 1978, Lu *et al.* 2023). A proposed mechanism for this loss of identity is the erosion of epigenetic structures that regulate gene expression. Structural elements such as chromatin normally help cells specialize by inhibiting or promoting gene expression depending on their placement on the DNA strand; however, they also play a role in DNA damage repair. As the need for repairing damage increases over time, chromatin loses its ability to regulate gene interactions, resulting in a loss of cell identity (Oberdoerffer *et al.* 2008, Lu *et al.* 2023).

**Figure 5:**
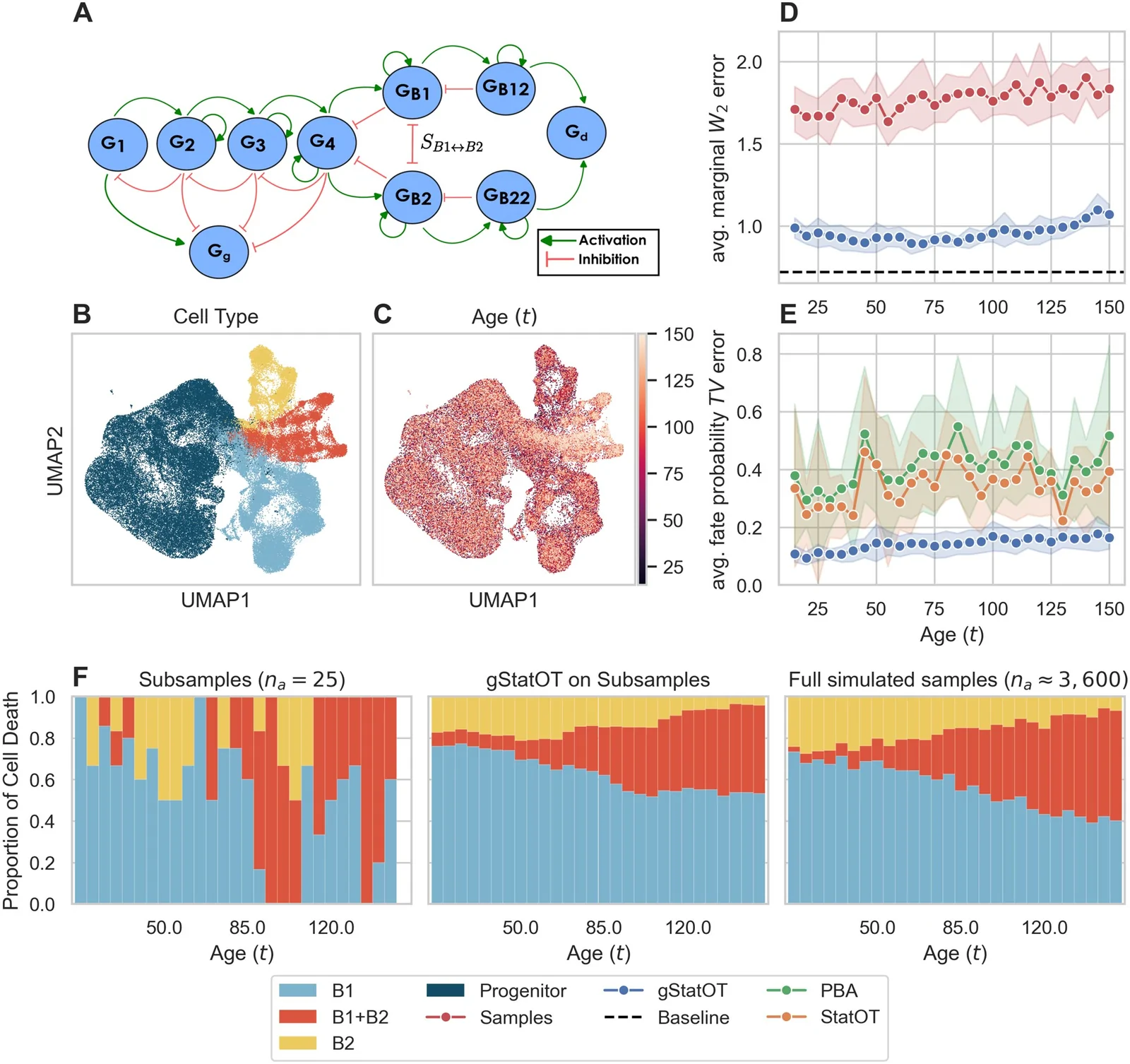
Simulated gene regulatory network (GRN) model for cell differentiation with progressive cell identity loss. (A) The GRN we used to simulate the loss of cell identity with age. Cells start out in a progenitor state defined by expression of $G_{1}$ and the proliferation gene $G_{g}$. They then progress into one of two lineages, B1 or B2, depending on which of the mutually repressive genes $G_{B1}$ or $G_{B2}$ they express. $S_{B1↔B2}$ represents the age-dependent strength of this repression. Upon terminally differentiating by expressing either of the lineage markers $G_{B12}$ or $G_{B22}$, cells begin to express $G_{d}$, which triggers apoptosis. (B, C) UMAP embedding of the simulated time course coloured by cell type and age, respectively. Note that the ambiguous B1 + B2 branch consists of cells primarily sampled at later ages. (D) Marginal distribution reconstruction performance for gStatOT on the downsampled data. (E) Fate probability reconstruction performance for gStatOT, StatOT, and PBA on the downsampled data. Results are summarized by the mean and standard deviation of each metric across 10 reseeded simulations. (F) Proportion of cells dying in each branch at each age as predicted by the sparse samples, gStatOT’s marginals, and the complete simulated data. See Fig. S1.3 for a quantitative comparison of these proportions across all ages.

To benchmark gStatOT on data from such a setting with an accessible ground truth, we developed a realistic simulation of a developmental system subject to progressive cell identity loss. We considered a branching system that develops according to the GRN in Fig. 5A, which specifies a chain of genes controlling both cell growth and differentiation. Cells start out in a progenitor state defined by expression of the proliferation gene, $G_{g}$, and the gene $G_{1}$. They then progress into one of two lineages: branch 1 (B1) or branch 2 (B2), depending on which one of the mutually repressive genes $G_{B1}$ or $G_{B2}$ they express. We biased cells towards B1 by specifying a larger interaction strength between $G_{4}$ and $G_{B1}$. Upon terminally differentiating, the cells begin to express $G_{d}$, which triggers apoptosis. To model the loss of cell identity with age, we made the repression strength, $S_{B1↔B2}$, between the lineage driver genes $G_{B1}$ and $G_{B2}$, decrease linearly with age. This can be viewed as cells in intermediate states inheriting deteriorated epigenetic structures that fail to inhibit one or the other gene (Kaufman and Rando 2010). As a result, cells are increasingly likely to terminate in an ambiguous state (denoted as B1 + B2) by expressing both lineage markers $G_{B12}$ and $G_{B22}$.

To realistically generate single-cell data from this system we employed the GRN simulator BoolODE that was developed as part of the BEELINE framework (Pratapa *et al.* 2020, Bravo González-Blas *et al.* 2023, McCalla *et al.* 2023). Since BoolODE does not simulate cell growth or allow for the regulatory strengths between genes to depend on time, we modified the stochastic ODE model it generated for our GRN by making the parameter $S_{B1↔B2}$ depend on age, and then proceeded to simulate it with birth and death, similar to what was done in our previous example. We periodically sampled from the simulation to create a time course with 28 ages. Ground truth fate probabilities for all sampled cells were obtained using the same method as in the previous example. To further emulate the noise in real sequencing data, we also followed BoolODE’s protocol for giving each sample in the time course a dropout rate of 50%. See SI.4.3 for the full details of our simulation procedure.

Figure 5B and C show the time course embedded in UMAP space (McInnes *et al.* 2018), coloured by cell type (e.g. branch B1, B2, or B1 + B2) and the age at which the sample was taken. Each cell type label was assigned based on each cell’s expression of the marker genes $G_{B12}$ and/or $G_{B22}$, after dropouts were added. As intended by our model, there is a mix of cells taken at all ages in branches B1 and B2, while the ambiguous B1 + B2 branch consists of cells primarily sampled at later ages.

We downsampled the simulated data to 25 cells per age before applying gStatOT to test its marginal distribution and fate probability reconstruction ability. The results of these tests are shown in Fig. 5D and E. Parameters for each of the methods tested were fit to optimize fate probability reconstruction (see SI.4.3 for application details and SI.7.2 for the parameter sweeps). We can see that gStatOT’s marginal distributions are again closer to the full simulated data at each age than the sparse samples—approaching the baseline distance between reseeded simulations. In Panel E we see that gStatOT improves fate probability reconstruction across all ages when compared with StatOT and PBA. In particular, it significantly reduces variance in the TV error across repeated runs compared to that of StatOT and PBA, with these latter methods suffering from the limited availability of sink states (i.e. cell states that express the lineage markers $G_{B12}$ and/or $G_{B22}$ and have negative growth rate due to their expression of $G_{d}$).

A property of potential biological interest in such systems is the proportion of cells dying in each state over the course of the aging process. Panel F illustrates these proportions, which were computed using the cell growth rates and each of the sparse samples, gStatOT’s marginals, and the complete simulated data. For example, with gStatOT, we compute the predicted (relative) number of cells dying in a branch *b* at age *a* as $\sum_{j}\left(1-e^{R(X_{j},a)\Delta t}\right)\mu_{a}(X_{j})$, where the sum is taken over sink cells, $X_{j}$, labeled as being a part of branch *b*. Here, we can see evidence that gStatOT’s constraint, enforcing mass balancing, is accurately correcting the sampling bias present in the sparse samples by placing mass on sink states so that the predicted proportion of cells dying in each of the branches better matches that of the ground truth. See Fig. S1.3 for a quantitative comparison of these proportions across all ages.

### 3.3 Human hematopoiesis

For an initial test of our method on real biological data, we applied gStatOT to a hematopoietic scRNA-seq time course that spans the entire human lifetime (Li *et al.* 2025). The time course consists of 22 time points: 8 gestational, taken between 10 and 23 weeks post-conception; 13 postnatal ages from donors aged 2 to 77; and umbilical cord blood samples. Each sample consists of hematopoietic stem and progenitor cells spanning a spectrum of differentiation states, from uncommitted to committed within 6 distinct lineages. Using this data set, Li *et al.* (2025) identified several age-specific changes in hematopoietic ontogeny. In particular, the independent application of StatOT at each age was used to measure the relative strength with which genes marked commitment to each lineage, which uncovered several age-related transcriptional programs. Here, we demonstrate that gStatOT improves trajectory reconstruction on sparse data by downsampling the samples at each age and comparing the results to age-independent analyses using StatOT and PBA.

Before applying these methods, we applied batch correction to the entire time course to establish smoother ground truth estimates (see SI.5 for details). Due to the significant change in timescale, from weeks to years, between the prenatal and postnatal ages, our model’s regularization coefficients are effectively 0 when bridging these time spans [see the discussion below (14)], and thus they do not encourage mixing them unless λ is chosen to be very large. So to avoid unnecessary computation time and allow for λ to be chosen appropriately for each scale, we chose to run our tests on the integrated data split into pre- and postnatal time courses, where the former runs from weeks 10 to 23 after conception, and the latter from birth to 77 years. We then established ground truth estimates for cell trajectories and fate probabilities by applying StatOT and PBA to the full samples at each age, following the same procedure for choosing parameters/growth rates used by Li *et al.* (2025) (see SI.5 for details). Each age was then downsampled before applying gStatOT and reapplying StatOT and PBA. gStatOT and StatOT’s estimates on the downsampled data were evaluated against the StatOT ground truth, while PBA was compared to its own ground truth.

On the sparse samples, parameters for each method were fit to a single downsampling of the data, using a weighted average of our marginal, trajectory, and fate probability metrics. See SI.7.3 for all parameter choices used in our evaluations. Each method’s performance was then evaluated on 10 repeated downsamplings of the full time courses.

**Figure 6:**
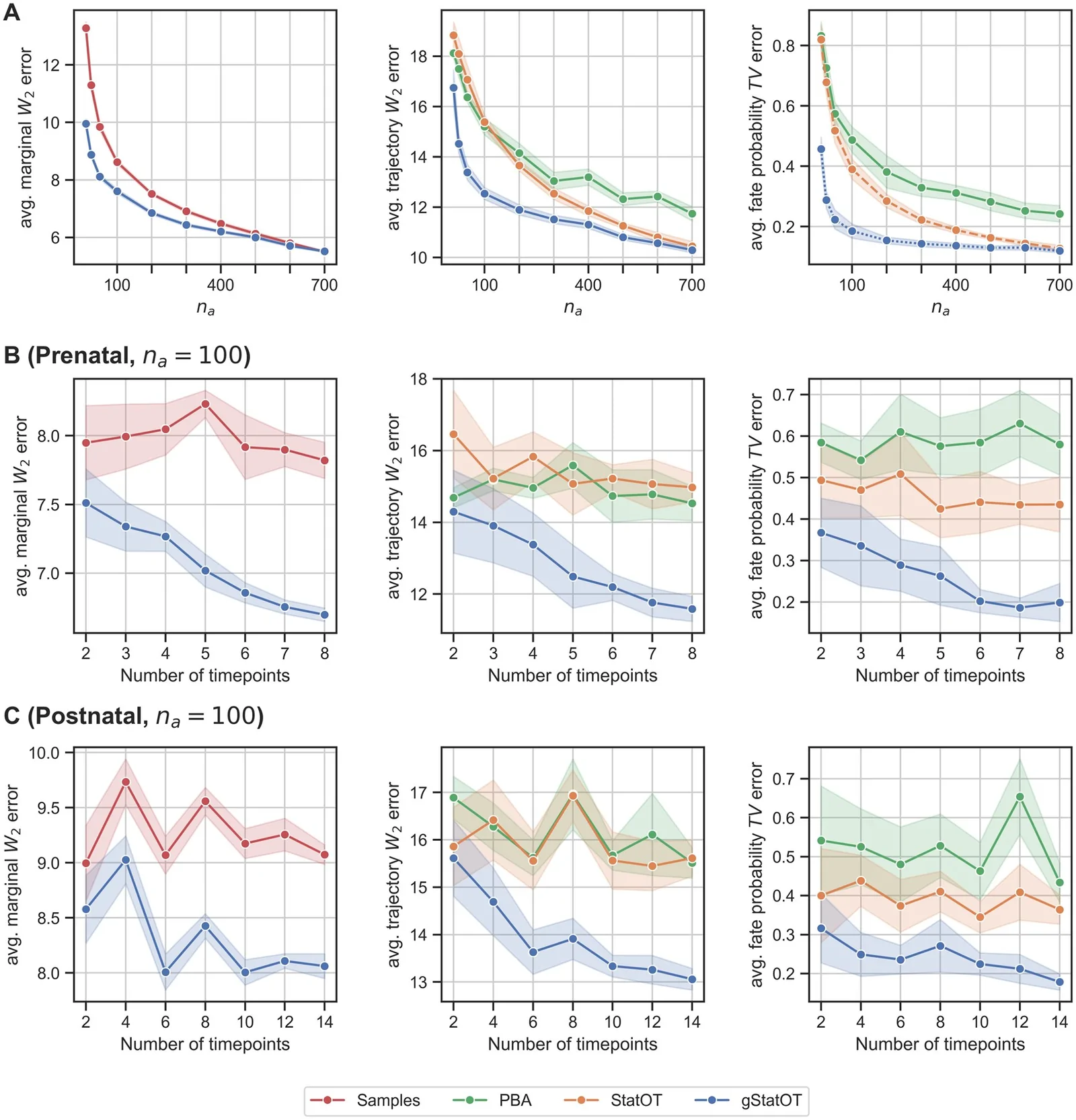
Average performance of each method using time courses with variable sample sizes (A) and temporal resolution (B and C), formed using the hematopoietic time course of Li *et al*. (2025). (A) Average performance of each method with varying numbers of cells per age, $n_{a}=10,25,50,100,\ldots ,700$. gStatOT took less than 2 minutes to solve on all levels of sparsity using a single NVIDIA H100 80GB HBM3 MIG instance (profile: 3 g.40gb). See Fig. S1.7 for the exact run times at each level. (B, C) Average performance of each method across all pre- and postnatal ages, when evaluated on time courses with varying temporal resolution, and a fixed number of cells per age ($n_{a}=100$). Results in all panels are summarized by the mean and standard deviation of each method’s average performance across age on 10 resamplings of the data.

Figure 6 shows the results of our tests on the downsampled time courses. Panel A displays the performance of each method across varying levels of sparsity. We observe that gStatOT is able to maintain an advantage over independent applications of StatOT and PBA in trajectory and fate probability estimation, across multiple levels of sparsity. Notably, it is able to reach the 0.2 TV error threshold with 4 times fewer cells than StatOT and at least 7 times fewer cells than PBA.

As with the simulated data, we tested gStatOT’s sensitivity to the number of ages present in the data. To form each time course, we held the initial and final times fixed and added intermediate samples to be as evenly spaced in time as possible. Parameters were tuned for each level of temporal resolution. The average of each metric across the pre- and postnatal time courses is displayed in Fig. 6B and C. We again observe that the performance of gStatOT improves with an increasing number of time points on both time courses, whereas the StatOT and PBA results remain flat, providing evidence that our method effectively exploits global information across all ages.

**Figure 7:**
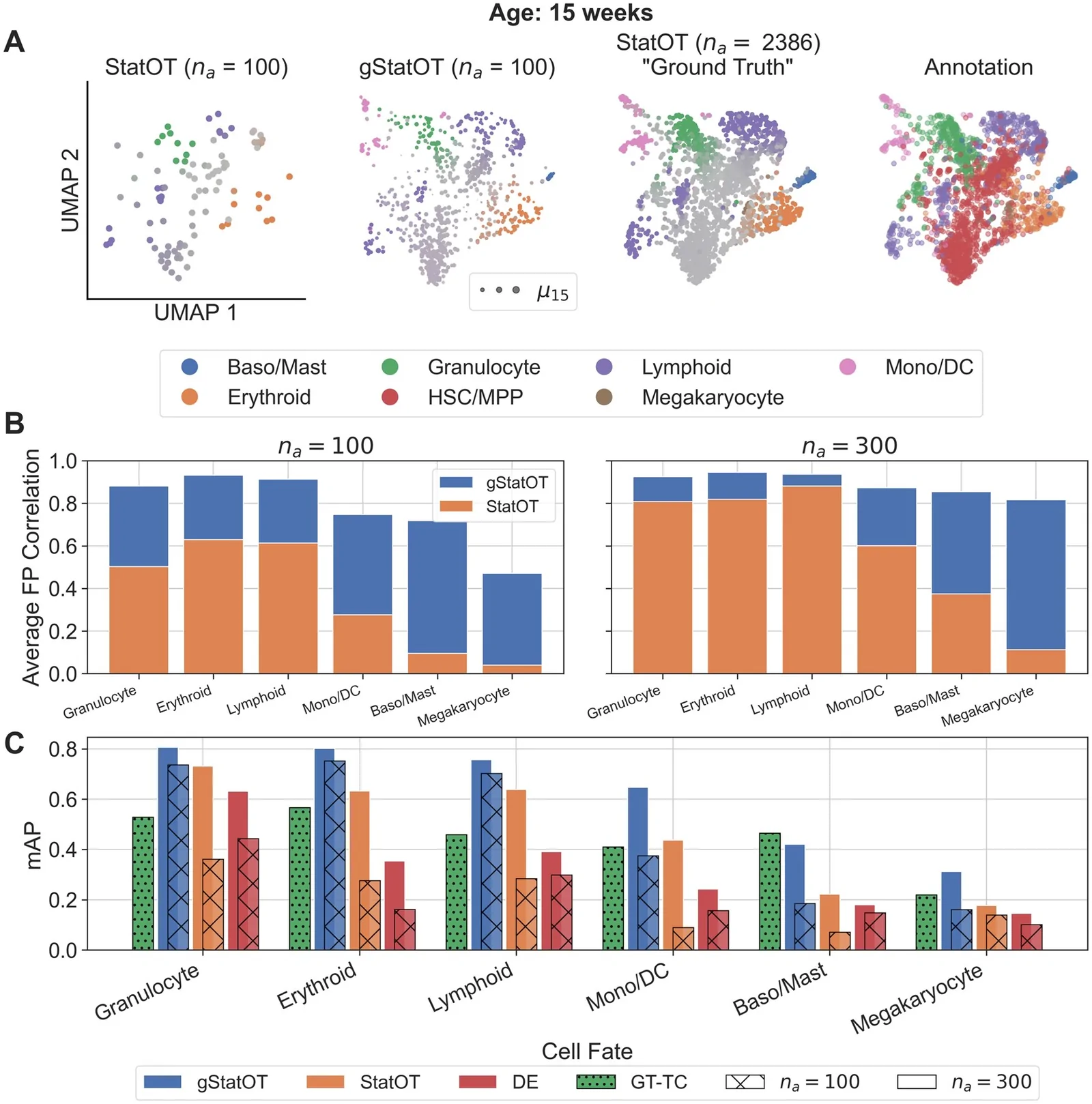
(A) Visualization of the week 15 sample in the integrated UMAP space, coloured by the predicted fate probabilities for each lineage and method, along with the cell type annotations derived by Li *et al*. (2025). Fate probabilities are saturated based on the strength of the predicted probability, e.g. the uncommitted HSCs are grey, while the cells predicted to be committed to the Mono/DC lineage are bright pink. The points in the gStatOT plot are scaled by the reconstructed marginals, $\mu_{i}$, to show gStatOT’s prediction for the relative number of cells in each state at this age. See Figs. S1.5–6 for similar plots for several ages that include each method’s inferred cell flow. (B) Correlation between the cells’ sparsely inferred fate probabilities and those obtained from the ground truth application of StatOT, averaged across all ages. (C) A measure of each method’s ability to identify and rank putative driver genes for each cell type across all ages. Each gene was ranked by the correlation between its expression and each lineage fate probability, obtained by gStatOT and StatOT on the sparse samples. The ranked lists at each age were then compared against the top 10 genes identified from our ground-truth StatOT estimates using average precision (AP). The models were evaluated on downsampled time courses with $n_{a}=100$ and 300 cells per time point, and the results are summarized by the mean AP (mAP) across all 22 ages. Two baselines are included: GT-TC is the mAP between consecutive ages’ ground-truth gene lists, representing the temporal consistency of these lists, and the DE baseline is the AP of the list of top differentially expressed genes between each cell type and the rest of the data set, at each age. Both baseline results are summarized by the mAP across all ages (see SI.6.4 for details).

To visualize the improved distribution and fate probability estimates obtained with gStatOT, in Fig. 7A we display a UMAP embedding of the integrated data coloured by the predicted fate probabilities for each lineage of the week 15 sample, alongside the cell type annotation done by Li *et al.* (2025). Fate probabilities are saturated based on the strength of the predicted probability, e.g. the uncommitted HSCs are grey, while the cells predicted to be committed to the Lymphoid lineage are a deep orange. Moreover, the points in the gStatOT plot are scaled by the reconstructed marginals, $\mu_{i}$. We can see that gStatOT’s marginals better match the full data distribution, and include the rarer Mono/DC and Baso/Mast lineages that are missing from the StatOT plot. Figure 7B shows the correlation between the cells’ sparsely inferred fate probabilities and StatOT’s ground truth estimates, averaged across all ages. We can see that gStatOT’s estimates are more strongly correlated with the ground truth over all lineages at the $n_{a}=100$ level of sparsity, and maintain this advantage on the less common, Baso/Mast and Megakaryocyte, lineages at the $n_{a}=300$ level.

A standard analysis downstream of TI is the identification of lineage driver genes. This is accomplished by ranking genes according to their correlation with the inferred lineage fate probabilities (Schiebinger *et al.* 2019, Weiler *et al.* 2024). So, we sought to test whether gStatOT’s improved fate probability estimates would translate into better identification of these sets of driver genes from sparse data. To do this, we generated a list of the top 10 putative driver genes for the cell lineages at each age by ranking them according to their expression’s correlation with each lineage fate probability predicted by our previous ground truth application of StatOT. We then evaluated the competency of gStatOT and StatOT in reconstructing these lists at each age from the downsampled time courses using average precision (AP) (Kishida 2005, Murphy 2022). Specifically, we computed the AP of each method’s gene list for each fate and age and reported the mean AP (mAP) across all ages in Fig. 7C (see SI.6.4 for details).

Our test was evaluated on downsampled data with 100 and 300 cells per time point, using the same model parameters that were fit during the previous evaluations. We observed that gStatOT achieved the best ranking of the top 10 relevant genes across all primary cell types, exhibiting an mAP comparable to or better than StatOT using only a third as many cells. However, both methods struggled on rare cell types such as Baso/Mast and Megakaryocyte. Since gStatOT’s fate probabilities maintain a relatively high correlation with those of the ground truth (Panel B) this is likely due to dropouts and sampling noise causing instability in the ground truth lists (measured by the green bar, which compares the ground truth lists at consecutive ages).

## 4 Discussion

We have presented our novel, theoretically motivated method, global StationaryOT, that reconstructs debiased cell trajectories from quasi-static aging single-cell snapshot time courses. We have shown, using both real and simulated data, that gStatOT can correct for data sparsity and provide significantly improved estimates compared to naive schemes that apply TI methods to samples independently. Moreover, the improved, globally supported cell-fate probability estimates enable more accurate identification of the genes involved in the GRN driving cell-fate specification.

Since gStatOT learns the data structure more effectively with increasing sampling rates, we hope it may open the possibility for new experimental designs that favour higher sampling rates over larger individual samples at each age. We speculate that future work on the convergence rate of gStatOT may show that such rapid sampling strategies are optimal when the total number of cells to be processed is fixed.

Our BCA implementation already enables the analysis of sizeable aging time courses, comprising hundreds of cells per time point. However, as we are solving multiple entropic optimal transport (EOT) problems, the method inherits a computational complexity that is quadratic in the number of cells, specifically $O(TN^{2})$. While this scaling has been manageable for only a single sample (Zhang *et al.* 2021), it presents a significant limitation when applied to time courses with tens of thousands of cells per sample. To address this, low-rank formulations of EOT have recently been used to scale OT-based TI methods to millions of cells (Klein *et al.* 2025), and a similar approach could be well suited to extend our method. Alternatively, warm-starting approaches could be explored to reduce the number of required iterations. One such approach could solve the problem on a reduced set of clustered cell states, after which the solution could be used to initialize the problem on the full data set.

Lastly, we note that in this work we have extended previous TI models and methods designed to infer cell trajectories that exclusively traverse gene expression space, and have modelled the effects of aging on these trajectories through the age-dependent epigenetic landscape Ψ. Being restricted to gene expression data prevents us from mechanistically modelling and inferring how epigenetic alterations in each cell drive the observed changes in gene expression and cell fate over age. Future work could extend our method to incorporate epigenetic data, such as chromatin accessibility with scATAC-seq (Baek and Lee 2020), which could not only improve the accuracy of our inferred trajectories, but also allow for the distributional changes in gene expression to be explained by the observed cell states and inferred cell dynamics at previous ages.

## Supplementary Material

btag532_Supplementary_Data

## Data Availability

An implementation of global StationaryOT is available at https://github.com/ColeBoyle/global-stationaryOT. All code for reproducing our results is archived on Zenodo at https://doi.org/10.5281/zenodo.20723235. The hematopoiesis data published by Li *et al.* (2025) is publicly available at https://www.ncbi.nlm.nih.gov/geo/query/acc.cgi? acc=GSE189161.
