# Supplementary material for: Global StationaryOT: trajectory inference for aging time courses of single-cell snapshots
Source: Bioinformatics. 2026 Jul 23;42(8):btag532. doi: 10.1093/bioinformatics/btag532 (PMC13466723; doi:10.1093/bioinformatics/btag532)
Supplement: Supplementary file 1 [file btag532_supplementary_data.pdf]

Supplementary information for Global StationaryOT: Trajectory inference for aging time courses of single-cell snapshots

Cole Boyle, Elias Ventre, Geoffrey Schiebinger

Contents

SI.1 Solving Global StationaryOT **2**
SI.1.1 Discretization of gStatOT 3
SI.1.2 Feasibility Condition and the Existence of a Unique solution 3
SI.1.3 Derivation and solving of the dual problem 4
SI.1.4 Implementation 5
SI.1.4.1 Block coordinate ascent solver 5

SI.2 Parameter selection **6**

SI.3 Fate probability estimation **7**
SI.3.1 Computing fate probabilities with flux rates 7
SI.3.2 Stabilizing fate probability estimation 7

SI.4 Simulation Details **8**
SI.4.1 General model application details 8
SI.4.2 Synthetic data: Aging epigenetic landscape 8
SI.4.3 Synthetic data: Loss of cellular identity over age induced by epigenetic erosion 10

SI.5 Hematopoiesis preprocessing and parameter selection **12**

SI.6 Evaluation Metrics **17**
SI.6.1 Marginal Error 17
SI.6.2 Trajectory Metric 17
SI.6.3 Fate probability metric 18
SI.6.4 Driver gene identification and average precision scoring 18

SI.7 Parameter sweeps **20**
SI.7.1 Synthetic data: Aging epigenetic landscape 20
SI.7.2 Synthetic data: Loss of cellular identity over age induced by epigenetic erosion 22
SI.7.3 Hematopoiesis 23
SI.7.3.1 Variable Sample Size 23
SI.7.3.2 Variable Number of Time Points 29

SI.8 Usage of LLMs **33**

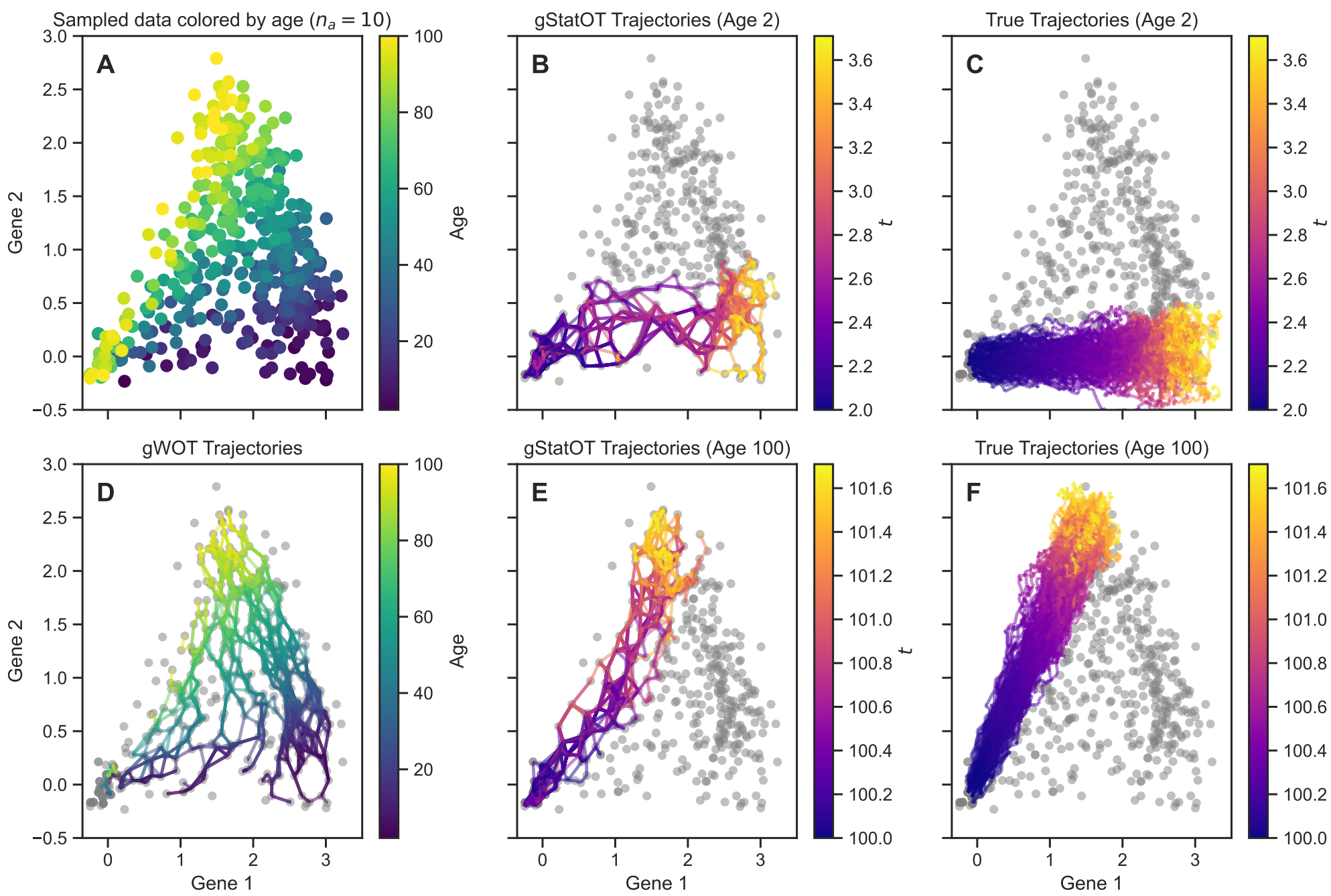

Figure SI.1: Comparison between trajectory inference with global WaddingtonOT (gWOT)[S1] and global StationaryOT (gStatOT) using time course data.
A: Data sampled from a simple single lineage, coloured by the age at which it was sampled. Cells start off in growing progenitor states near the origin, and then develop by expressing gene 1. As the system ages, cells begin to express increasing amounts of gene 2 during development. **B:** Cell trajectories inferred by gStatOT at age 2, using all the sampled data. These match the true cell trajectories depicted in Panel **C**, both in that they primarily involve only gene 1 and that they terminate in around 1.6 chronological time units (t). **D:** Since gWOT assumes the samples in the time course are the temporal marginals of cell trajectories, the cell paths that it infers traverse the entire aging spectrum. These paths start in the initial, age 2, sample and end in the final, age 100, sample. This makes them perpendicular to the true trajectories, and moreover fails to capture the aging effect in the system. **E,F:** In contrast to gWOT, gStatOT leverages the cell growth rates to infer the correct directionality of the paths, while simultaneously utilizing the time course data to infer trajectories that better emulate the ground truth cell paths at every sampled age. The code for generating this simulation and figure can be found at `https://github.com/ColeBoyle/global-StationaryOT/extra/gWOT_comparison`.

SI.1 Solving Global StationaryOT

In this section, we show that the discretized version of gStatOT has a unique solution, provided that our cell growth rates satisfy the natural condition that sources and sinks are specified at every age. We then show how we derive and solve the corresponding dual problem.

SI.1.1 Discretization of gStatOT

Recall that we approximate the full cell state space by the union of our samples, $\overline{\mathcal{X}}$. Letting $N = |\overline{\mathcal{X}}|$ be the total number of sampled cells, with this approximation, $\mathcal{P}(\overline{\mathcal{X}} \times \overline{\mathcal{X}})$ becomes the set of nonnegative $N \times N$ matrices with unit sum whose rows and columns are indexed by the elements of $\overline{\mathcal{X}}$. Using this discrete approximation of the state space, our recovery program may be written as

$$\begin{aligned} &\inf_{\overline{\pi} \in (\mathbb{R}^{N \times N})^T} \lambda \mathbf{Reg}(\overline{\pi}; \varepsilon_1) + \mathbf{Fit}(\overline{\pi}; w, \varepsilon_2, \varepsilon_3) \\ &\text{such that } \pi_i \mathbb{1}_N = \hat{g}_i \odot (\pi_i)^\top \mathbb{1}_N \\ &\qquad\qquad \mathbb{1}_N^\top \pi_i \mathbb{1}_N = 1 \\ &\qquad\qquad \pi_i \geq 0 \\ &\qquad\qquad \forall i = 1, ..., T \end{aligned} \tag{Discretized gStatOT}$$

where $\mathbb{1}_N$ is the vector of 1's of length N. To make the problem easier to solve, we approximate the OT problems in **Fit** and **Reg** by their entropic version, with regularization strength $\varepsilon_1 = \varepsilon_3 = 5 \cdot 10^{-3}$. See Section SI.2 for more details on selecting the parameters $\lambda, w, \Delta t$, and ε_2.

SI.1.2 Feasibility Condition and the Existence of a Unique solution

Our constraint, which forces a balancing of growth at each age, introduces the feasibility condition that we must have both sources and sinks at each age, or else have neither. gStatOT can naturally satisfy this condition by sharing growth rate information across the time course. Here, we show that this condition is not only necessary but also sufficient for the existence of a unique solution to the discretized gStatOT problem.

Our constraint set is compact and convex (the intersection of a linear and convex constraint, with the unit sum constraint ensuring boundedness), and our objective function is strictly convex and continuous. In order to show the existence and uniqueness of solutions to gStatOT, it suffices to show that this constraint set is non-empty. This occurs exactly when our global sample specifies both source and sink cells at each age. That is, for each $i = 1, ..., T$, $\hat{R}_i$ must satisfy the following

$$\text{There exists } j \text{ such that } \hat{R}_{ij} > 0 \text{ if and only if there exists } k \text{ such that } \hat{R}_{ik} < 0.$$

We note that this also applies to the situation where we have neither sources nor sinks at a given age; however, this case is less interesting since it predicts no cell movement. Moreover, it is unlikely to occur in applications.

Proof. Fix some i and let $\hat{g}_{ij} = e^{\hat{R}_{ij}\Delta t}$, $\mathbb{1} = [1, 1, ..., 1]^\top$. In the discrete setting, our constraints can be written as

$$\begin{aligned} \pi_i \mathbb{1} &= \hat{g}_i \odot \pi_i^\top \mathbb{1} \\ \mathbb{1}^\top \pi_i \mathbb{1} &= 1 \\ \pi_i &\geq 0 \\ i &= 1, ..., T \end{aligned}$$

Let $b_i = \pi_i^\top \mathbb{1}$. Then there exists a π_i satisfying the constraints if and only if there exists b_i such that $\sum_j b_{ij} \hat{g}_{ij} = \sum_j b_{ij} = 1, b_{ij} \geq 0$. That is, there must exist a positive solution to the following linear system

$$\begin{bmatrix} \mathbb{1} \\ \hat{g} - \mathbb{1} \end{bmatrix} b_i = \begin{bmatrix} 1 \\ 0 \end{bmatrix} \tag{1}$$

Farkas' lemma [S2] implies that b_i exists if and only if there does not exist $y_1, y_2 \in \mathbb{R}$ such that

$$\begin{aligned} y_1 &< 0 \\ y_1 + y_2(\hat{g}_{ij} - 1) &\geq 0, \forall j \end{aligned}$$

Clearly, if $\hat{g}_i > 1$ we may choose $y_2 = 1$ and $-\min_j(\hat{g}_{ij} - 1) < y_1 < 0$, and a similar situation exists if $\hat{g}_i < 1$. Whereas, if there exists j, l such that $\hat{g}_{ij} < 1$ and $\hat{g}_{il} > 1$ then we would need

$$\begin{aligned} y_1 + y_2(\hat{g}_{ij} - 1) \geq 0 &\implies y_2 \leq -\left|\frac{y_1}{\hat{g}_{ij} - 1}\right| \\ y_1 + y_2(\hat{g}_{il} - 1) \geq 0 &\implies y_2 \geq \left|\frac{y_1}{\hat{g}_{il} - 1}\right| \end{aligned}$$

These conditions would imply that $y_2 = 0$, but y_2 can't be 0 since this would contradict the first condition that $y_1 < 0$. Finally, if $\hat{g}_i \equiv 1$, the conditions above imply that $y_1 < 0$ and $y_1 \geq 0$, thus no solutions exist. Therefore, Farkas' lemma implies that a non-negative b_i solving Equation 1 exists if and only if our stated condition holds. □

SI.1.3 Derivation and solving of the dual problem

Converting each of the OT problems in **Reg** and **Fit** to their dual forms and converting our growth constraint into a Lagrange multiplier, we may write the discretized gStatOT problem as

$$\begin{aligned} \inf_{\bar{\pi} \in \mathcal{P}(\bar{\mathcal{X}} \times \bar{\mathcal{X}})^T} \sup_{\phi_i, \psi_i, \gamma_i, \varrho_i \in \mathbb{R}^N, f_i \in \mathbb{R}^N} & \sum_i^{T-1} \left(\langle \phi_i, \pi_i^\top \mathbb{1} \rangle + \langle \psi_i, \pi_{i+1}^\top \mathbb{1} \rangle - \mathrm{OT}^*_{\varepsilon_1}(\phi_i, \psi_i) \right) \\ & + \sum_i^T \left(\mathcal{H}_{\varepsilon_2}(\pi_i) + w(\langle \gamma_i, \pi_i^\top \mathbb{1} \rangle + \langle \varrho_i, \hat{\rho}_i \rangle - \mathrm{OT}^*_{\varepsilon_3}(\gamma_i, \varrho_i)) \right) \\ & - \sum_i^T \langle f_i, \pi_i \mathbb{1} - \hat{g}_i \odot (\pi_i^\top \mathbb{1}) \rangle \end{aligned} \tag{2}$$

where

$$\mathrm{OT}^*_\varepsilon(a, b) = \varepsilon \sum_{ij} e^{a_i/\varepsilon} e^{-C_{ij}/\varepsilon} e^{b_j/\varepsilon} \tag{3}$$

is the Legendre transform of OT_ε. Let $\tilde{\mathcal{F}}$ denote the objective function in (2). Since $-\mathrm{OT}^*_\varepsilon$ is jointly concave in its arguments, $\tilde{\mathcal{F}}$ is concave in $\phi, \psi, \gamma, \varrho$ for fixed $\bar{\pi}$. Moreover, $\tilde{\mathcal{F}}$ is convex in $\bar{\pi}$ when all other arguments are fixed since $\mathcal{H}_\varepsilon$ is convex and the maps $\pi \mapsto \langle a, \pi \mathbb{1} \rangle, \pi \mapsto \langle a, \pi^\top \mathbb{1} \rangle$ are linear for all $a \in \mathbb{R}^N$. Thus, since $\mathcal{P}(\bar{\mathcal{X}} \times \bar{\mathcal{X}})$ is convex and compact, we may apply Sion's minimax theorem to swap the inf and sup

$$\begin{aligned} & \inf_{\bar{\pi} \in \mathcal{P}(\bar{\mathcal{X}} \times \bar{\mathcal{X}})^T} \sup_{\phi_i, \psi_i, \gamma_i, \varrho_i, f_i \in \mathbb{R}^N} \tilde{\mathcal{F}}(\bar{\pi}, \phi, \psi, \gamma, \varrho, f) \\ & \quad = \sup_{\phi_i, \psi_i, \gamma_i, \varrho_i, f_i \in \mathbb{R}^N} \inf_{\bar{\pi} \in \mathcal{P}(\bar{\mathcal{X}} \times \bar{\mathcal{X}})^T} \tilde{\mathcal{F}}(\bar{\pi}, \phi, \psi, \gamma, \varrho, f) \end{aligned}$$

The last step is a standard application of the Fenchel-Rockafellar (FR) duality theorem to remove our final constraint. Let A be the linear map that sends $\bar{\pi} \in (\mathbb{R}^{N \times N})^T$ to the sums of its elements, $A\bar{\pi} = [\mathbb{1}^\top \pi_1 \mathbb{1}, ..., \mathbb{1}^\top \pi_T \mathbb{1}]^\top \in \mathbb{R}^T$. Letting $\chi_{\mathbb{1}_T}$ be the indicator for the vector of ones of length T,

and noting that $\tilde{\mathcal{F}}$ is only finite when $\bar{\pi} \geq 0$ (and we know such a solution exists because of the previous result), we may write

$$\inf_{\bar{\pi}\in\mathcal{P}(\bar{\mathcal{X}}\times\bar{\mathcal{X}})^T} \tilde{\mathcal{F}}(\bar{\pi},\phi,\psi,\gamma,\varrho,f) = \inf_{\bar{\pi}\in(\mathbb{R}^{N\times N})^T} \tilde{\mathcal{F}}(\bar{\pi},\phi,\psi,\gamma,\varrho,f) + \chi_{\mathbb{1}_T}(A\bar{\pi})$$

Next, holding all dual variables fixed, the Legendre transform of $\tilde{\mathcal{F}}$ and $\chi^*_{\mathbb{1}_T}$ is given by

$$\tilde{\mathcal{F}}^*(H,\phi,\psi,\gamma,\varrho,f) = \sup_{\bar{\pi}\in(\mathbb{R}^{N\times N})^T} \langle H,\bar{\pi}\rangle - \tilde{\mathcal{F}}(\bar{\pi},\phi,\psi,\gamma,\varrho,f),\ \ H\in(\mathbb{R}^{N\times N})^T$$

$$\chi^*_{\mathbb{1}_T}(h) = \sup_{x\in\mathbb{R}^T} \langle h,x\rangle - \chi_{\mathbb{1}_T}(x) = \langle h,\mathbb{1}\rangle,\ \ h\in\mathbb{R}^T$$

Now we can apply FR [S3, Theorem 4.4.3],

$$\begin{aligned}
&\inf_{\bar{\pi}\in(\mathbb{R}^{N\times N})^T} \tilde{\mathcal{F}}(\bar{\pi},\phi,\psi,\gamma,\varrho,f) + \chi_{\mathbb{1}_T}(A\bar{\pi}) \\
&\qquad = \sup_{h\in\mathbb{R}^T} -\tilde{\mathcal{F}}^*(A^\top h,\phi,\psi,\gamma,\varrho,f) - \chi^*_{\mathbb{1}_T}(-h) \\
&\qquad = \sup_{h\in\mathbb{R}^T} \inf_{\bar{\pi}\in(\mathbb{R}^{N\times N})^T} \tilde{\mathcal{F}}(\bar{\pi},\phi,\psi,\gamma,\varrho,f) - \langle h, A\bar{\pi} - \mathbb{1}_T\rangle
\end{aligned} \tag{4}$$

The inner minimization problem is solved by simply setting the gradient of $\tilde{\mathcal{F}}$ in $\bar{\pi}$ to zero, holding all else fixed. By doing so, we obtain the optimal $\pi_i^\star$

$$\begin{aligned}
(\pi_i)^\star_{kl}(\phi,\psi,\gamma,f,h) &= \exp\left(-\frac{\lambda(\phi_{il}+\psi_{(i-1)k}) + C_{kl} + w\gamma_l - (f_{ik} - \hat{g}_{il}f_{il}) - h_k}{\varepsilon_2}\right) \\
& i = 1,\dots,T, \\
& \phi_T := 0, \psi_0 := 0
\end{aligned} \tag{5}$$

Substituting $\bar{\pi}^\star$ into (4), we obtain the dual problem of discrete gStatOT

$$\sup_{\phi_i,\psi_i,\gamma_i,\varrho_i,f_i\in\mathbb{R}^N,h\in\mathbb{R}^T} \tilde{\mathcal{F}}(\bar{\pi}^\star(\phi,\psi,\gamma,f,h),\phi,\psi,\gamma,\varrho,f) - \langle h, A\bar{\pi}^\star(\phi,\psi,\gamma,f,h) - \mathbb{1}_T\rangle \tag{6}$$

$$:= \sup_{\phi_i,\psi_i,\gamma_i,\varrho_i,f_i\in\mathbb{R}^N,h\in\mathbb{R}^T} \mathcal{D}(\phi,\psi,\gamma,\varrho,f,h) \tag{7}$$

SI.1.4 Implementation

SI.1.4.1 Block coordinate ascent solver

The total gradient of $\mathcal{D}$ can be manually computed, and off-the-shelf gradient based solvers can be used to solve (7). However, this requires evaluating the exponential in (5), which can lead to numerical instability if ε_2 is small. Instead, analogous to the algorithm used to stabilize traditional entropic OT problems [S4], we use a block coordinate ascent (BCA) approach [S5] that allows us to exploit the log-sum-exp trick to stabilize exponential evaluations.

In particular, we update each block of dual variables, $\phi,\psi,\gamma,\varrho,f,h$, sequentially by maximizing $\mathcal{D}$ with respect to the block being updated, while holding all other blocks fixed. The updates for ϕ,ψ,γ,ϱ and h are given by closed-form expressions, as $\nabla_z\mathcal{D} = 0$ can be solved analytically for

$z \in \{\phi, \psi, \gamma, \varrho, h\}$*. All of these expressions can exploit the log-sum-exp trick to avoid evaluating exponentials. However, the update for f is not given by a closed-form expression, since $\nabla_{f_i}\mathcal{D}$ is equal to our growth constraint and $\pi_i^\star \mathbb{1} - \hat{g}_i \odot ((\pi_i^\star)^\top \mathbb{1}) = 0$ is a highly coupled set of nonlinear equations in f_i. Therefore, we update f by performing a single step of the LBFGS algorithm [S6] on $\log(\sum_i \pi_i^\star)$ with respect to f. This step is equivalent to maximizing $\mathcal{D}$ with respect to f, but allows us to again exploit the log-sum-exp trick to stabilize the computation.

We repeat this process until convergence, which we determine by evaluating the relative duality gap. If $\mathcal{D}$ is the dual objective and $\mathcal{P}$ is the primal objective, we compute the duality gap as $G(\mathcal{P}, \mathcal{D}) = \mathcal{P} - \mathcal{D}$, and the relative duality gap as $RG(\mathcal{P}, \mathcal{D}) = \frac{G(\mathcal{P},\mathcal{D})}{\mathcal{P}+\mathcal{D}+1}$. We stop once $RG(\mathcal{P}, \mathcal{D}) < 10^{-3}$, which we find to be sufficient for convergence in practice. Computing the objectives does require evaluating the exponential in (5), so we only check for convergence every 100 iterations and proceed even if the duality gap is not computable, as early instability in these evaluations doesn't affect the rest of the algorithm.

We implemented this algorithm in Python using the JAX library [S7] and used the optax [S8] implementation of LBFGS for updating f, allowing it to be efficiently executed on GPUs. The code for this implementation can be found at `https://github.com/ColeBoyle/global-StationaryOT`.

SI.2 Parameter selection

gStatOT has four main parameters: λ, which controls the between sample regularization strength; w, which controls the strength of the fit to the sampled data at each age; ε_2, which controls the strength of the entropic regularization in **Fit**; and Δt, which controls the time step of our splitting approximation and represents the timescale at which we expect cells to change state.

w**:** We find that on sparse data gStatOT is robust to the choice of w, and so we simply set it to 1 for most of our analyses. As the sample size increases, to match the dynamics inferred by StatOT we must increase w. For example, with the $n_a = 500, 600$, and 700 time courses in Figure 6, we set w to 10.

λ**:** The choice of λ depends on the sampling rate and sample size, and so must be tuned based on the sparsity of the specific dataset. λ should decrease with increasing sample size and sampling rate. However, as noted in the text, λ contains an implicit estimate of the aging rate, $\lambda \sim 1/c$, and so it may be desirable to choose λ larger if the aging rate is expected to be slow, regardless of the sampling rate. In practice, we find that an initial value of λ between 5 and 20 works well for our tested datasets, where our quantitative results were relatively stable across this range.

ε_2**:** Based on the connection between diffusion processes and optimal transport, ε_2 is theoretically equal to $\sigma^2 \Delta t$. In practice, we simply take ε_2 to represent the expected noise in the data and tune or choose Δt separately. A common heuristic for finding this noise level is to adjust it so that the total predicted fate probability for each lineage matches the proportion of cells previously identified to be a part of that lineage (e.g., with clustering and marker genes) [S9, S10], see Figure SI.4 for an example of this approach for choosing ε using StatOT and PBA's diffusion parameter D.

Δt**:** Since our splitting approximation improves with decreasing Δt, it is ideally chosen to be as small as possible. However, this can lead to overly flat cell growth, $\hat{g}_i$, which disincentivizes cell movement. So we recommend taking it to be large enough so that cell trajectories converge to sink states within a reasonable number of iterations. The value 0.25 worked well as a default value for StatOT, and we used this value for our hematopoiesis study.

*∇_z denotes the gradient with respect to z

SI.3 Fate probability estimation

SI.3.1 Computing fate probabilities with flux rates

To compute the fate probabilities of cells, we expand upon the method used in the original StatOT paper [S10], by incorporating the flux rate of cells dying or leaving the system instead of simply treating every sink cell as an absorbing state.

We use a similar approach to that of Weinreb et al. [S9]. In particular, for each of the K lineages or cell types, l_k, that are present in our system, we build a sink matrix $S \in \mathbb{R}^{N \times K}$, where N is the number of cell states and for cells labeled as being part of lineage l_k, we take S_{jk} to be the probability that the cell X_j^a leaves the system as a type l_k within the period of time Δt, namely, $|\min\{R(X_j^a, a), 0\}|\Delta t$. After row-normalizing our couplings π_i we augment them with S to form a new transition matrix, P_i, that describes the probability of transitioning from one cell state to another, or leaving the system in a particular lineage over the period of time Δt. In particular, we have

$$P_i = \begin{pmatrix} \pi_i & S \\ 0 & I \end{pmatrix} \tag{8}$$

After another row normalization, we interpret P_i as the transition matrix of a Markov chain, and proceed to compute the fate probabilities of cells by solving for the absorption probabilities of this Markov chain as previously done (see supplementary section 1.2 of [S10]).

SI.3.2 Stabilizing fate probability estimation

As with other methods that estimate cell dynamics with Markov chains [S9, S11, S10] our ability to estimate the cell dynamics does not always imply that we are able to stably compute cell fate probabilities. For the OT based methods, this occurs when one wishes to use small ε values which can lead to sparse transition matrices, and thus disconnected cell states that are unable to reach any of the sink states. In practice, this can occur if the data contains outlier cell states that are predicted to be rare and reachable only by fully random motion and not the natural flow of cells. In this case, the resulting transition matrices may be ill-conditioned for solving for cell fate probabilities.

Other methods [S11, S10] have addressed this limitation by increasing the base noise level by perturbing the transition probabilities a small amount after they have already been fit to the data. Here, we employ an alternative method that seeks to remove these outlier states before computing the fate probabilities for the rest of the cells. In particular, if X_j is an outlier state, then little to no mass should flow through this state, hence, our inferred coupling π will assign very low probabilities to points (X_j, X_k) or (X_k, X_j) for $k \neq j$. That is, setting mass $\text{in}_j = \sum_k \pi_{kj} - \text{diag}(\pi)_j$ and mass $\text{out}_j = \sum_k \pi_{jk} - \text{diag}(\pi)_j$, we will have that mass $\text{flow}_j =$ mass $\text{out}_j +$ mass in_j is very small. Thus, we can detect and remove outlier states by simply pruning states with low mass flow. To implement this, we compute a mass flow distribution by computing mass flow_j for each cell state[†], and then remove those states with mass flow below the q-th percentile of this distribution. q is initially chosen to be 0, but if we fail to compute fate probabilities due to ill-conditioning or poor convergence, we increase q by 0.01 and repeat this process until we are able to compute fate probabilities, or until q reaches a maximum value of 0.1. In practice, we find that this method allows us to stably compute fate probabilities for a wider range of ε values, and thus obtain more accurate estimates of cell dynamics when the overall noise level in the data is low, but our samples still contain outliers. We employ this strategy when computing fate probabilities with both gStatOT and StatOT.

[†] We double the mass flow of sink states since they have naturally less out flow

Lastly, we note that this only works when using the coupling π. Once π is row-normalized to form the transition matrix we lose the ability to compare the mass flow of different states.

SI.4 Simulation Details

SI.4.1 General model application details

gStatOT and StatOT: For both our simulations, gStatOT and StatOT's Δt parameter were set to the simulation time step dt. Fate probabilities were computed as described in Section SI.3. The StatOT algorithm was implemented using the Optimal Transport Tools (OTT) Python library [S12]. In order to provide the best possible competitor to gStatOT, we used the log-domain stabilized version of the Sinkhorn algorithm implemented in OTT, which provides greater stability in low ε settings. For greater stability, both methods were given cost matrices scaled by the mean of their entries. Moreover, both methods were given the true growth rates of cells at the age they were sampled. For an analysis of the sensitivity of both methods to growth rate estimation error, see Figure SI.2. Other parameters were selected via the parameter sweeps described below.

PBA: To apply PBA [S9], we ran the `PBA_pipeline.py` script provided by the authors at https://github.com/AllonKleinLab/PBA. We made slight modifications to the script to allow us to run it within our testing framework, mainly to make sure it didn't crash when it failed to compute fate probabilities at a particular age. In such cases, we reported the fate probability metric as 1, which is the maximum value it can take. We also modified the script to allow us to save the transition matrices that PBA constructs so that we could sample individual cell trajectories to use for our trajectory metric. As with gStatOT and StatOT, we gave PBA the true growth rates of cells at the age they were sampled. We set the lineage specific flux rates, $S^a \in \mathbb{R}^{N\times K}$, that PBA uses to compute fate probabilities, to be the death rate of sink cells in that particular lineage, i.e., $S^a_{ij} = \min\{0, -R(X^a_i, a)\}$ if X_i is a part of lineage l_j. The value of k used to construct the k-NN graph, and diffusion level D, that PBA uses to construct its transition matrices were selected via the parameter sweeps described below. The exact code we used is archived at https://doi.org/10.5281/zenodo.20723235.

SI.4.2 Synthetic data: Aging epigenetic landscape

For our first simulation, we generated a system that evolves according to Equation 3 in the main text. We used the potential

$$\Psi(X,t) = \frac{1}{2}\|z_0(ct) - X\|^2\|z_1(ct) - X\|^2 \tag{9}$$

where $c = 5 \times 10^{-3}$/day is our aging rate, and z_0, z_1 are well locations

$$z_0(t) = [1, 1.25, 0, ..., 0]^\top + t[1, 0, 1, 0, ..., 0]^\top$$
$$z_1(t) = [1, -1.25, 0, ...0]^\top + t[1, 0, 1, 0, ..., 0]^\top$$

representing the location of the terminal cell states at the age $a = ct$. The following cell division rate, α, and death rate, β, were used

$$\alpha(X,t) = \left(\begin{cases} 100, & \text{if } X \equiv 0 \\ ctX_2, & \text{if } X_2 > 0, \text{ and } \|X - z_0(ct)\| > 0.3 \\ 0, & \text{otherwise} \end{cases}\right) /\text{day} \tag{10}$$

$$\beta(X,t) = \left(\begin{cases} 5, & \text{if } \|X - z_0(ct)\| < 0.3 \text{ or } \|X - z_1(ct)\| < 0.3 \\ 0, & \text{otherwise} \end{cases}\right) /\text{day} \tag{11}$$

Lastly, the diffusion coefficient was taken to be $\sigma^2 = 0.1$.

To generate an aging time course from this system, we started with a population of 5 fixed cells (which do not feel the effects of drift or diffusion) located at the origin, and simulated this system for 99 days using the Euler-Maruyama method with a time step of $dt = 0.01$ days. Division and death were applied at each iteration by adding (if $R(x,a) = \alpha(x,a) - \beta(x,a) > 0$) or removing (if $R(x,a) < 0$) a cell at position x and age t, with probability $|R(x,a)|dt$. We generated an aging time course by sampling all cells from the simulation every day starting at day 2. This time course was then subset using the various sampling rates listed in Figure 4.

For each sample in the time course, a ground truth distribution of cell trajectories was generated by simulating 500 individual cell trajectories, each starting from a cell sampled with weight proportional to its division rate, $\max\{R(x,a), 0\}$. Each trajectory was run until the cell died, revealing an empirical estimate that the average cell lifespan was approximately 3.5 days at day 2 and decreased to 2 days at day 99, due to the increase in shorter cell trajectories resulting from the growth increase in the +2 lineage. So we truncated all trajectories to 2 days for comparison with each method's reconstructed trajectories.

Estimates of the ground truth fate probabilities were obtained by simulating 500 trajectories starting from each sampled cell and computing the fraction of trajectories that ended in the +2 and −2 lineages, respectively.

Time courses with each of the specified sampling rates in Figure 4 were downsampled to $n_a = 10$ cells per age before applying gStatOT to the full time course and the methods StatOT and PBA to each individual time point.

For each sampling rate, we performed a sweep over each method's parameters and selected those that minimized the mean of the marginal, trajectory, and fate probability metrics (see Section SI.6 for a detailed discussion of these metrics). To evaluate the trajectory metric, 2 day long (i.e. 200 step) cell paths were sampled from each of the method's transition matrices. Before taking the mean over all the metrics, we scaled each metric by its minimum and maximum values over all method and parameter choices. As discussed in Section SI.3, certain parameter values may lead any of the methods to produce transition matrices that are ill-conditioned for computing fate-probabilities; moreover, the lack of sink states at some ages makes it impossible for StatOT and PBA to compute these probabilities. If a method failed to compute fate probabilities at a certain age we set the total variation metric to its maximum value of 1. Trajectories that were shorter than 2 days were padded with their final state to reach the length required for comparison.

The mean metric and optimal parameters for each method and sampling rate are shown in Section SI.7.1. The parameters used for the results depicted in Figures 3 and 4 in the main text are highlighted in red. If a method failed to produce fate probabilities at any age for that parameter choice we highlighted the mean metric value in orange.

To produce the results in Figure 4, the methods were run using the optimal parameters for each sampling rate, on 10 reseedings of the simulation and time course generation process. The results were summarized by taking the mean and standard deviation of each metric across the 10 runs.

Code for generating the simulation data and figures 3 and 4 in the main text can be found at https://github.com/ColeBoyle/global-StationaryOT/.

SI.4.3 Synthetic data: Loss of cellular identity over age induced by epigenetic erosion

To develop a simulation that more closely resembles a real aging developmental system, we employed the gene regulatory network (GRN) simulator BoolODE [S13], which simulates realistic scRNA-seq data resulting from the dynamics of a user-specified Boolean GRN with specified gene interaction strengths. However, BoolODE does not support simulating either cell growth or time dependent interaction strengths, and so we used BoolODE to translate the GRN in Figure 5 Panel A into a stochastic ODE model and modified the simulation to support both of these components.

In more detail, BoolODE takes a Boolean GRN and a list of gene interaction strengths, S, as input and outputs a model for cell velocities in the gene and protein expression spaces. Specifically, letting a cell state be denoted by $X_t = (G_t, P_t)$, where G_t denotes the list of genes and P_t the list of associated proteins, BoolODE outputs a velocity vector, $V(X_t, t, S)$, that describes the deterministic part of the dynamics of X_t. Normally, BoolODE would then simulate cell trajectories evolving according to the SDE

$$dX_t = V(X_t, t, S)dt + \sqrt{X_t}dW_t \tag{12}$$

with boundary conditions to ensure that gene and protein expression remain non-negative.

To simulate the loss of cellular identity over age that results from epigenetic erosion, we modified the regulation strength, $S_{B1\leftrightarrow B2} \in S$, between the lineage driver genes, G_{B1} and G_{B2}, so that it decreased linearly with age from 0.9 at age 0 to 0.5 at age 150. We incorporated cell growth by giving cells rates of division and death that depended on the abundance of the proteins P_g and P_d, that are coded for by the growth gene G_g and apoptosis gene G_d respectively. Specifically, cells with more than 15 units of P_g were given a division rate of 2 per (chronological) age, while cells with more than 15 units of P_d were given a death rate of 1 per age.

With our modified S, which now depends on age, we simulated (12) for 150 ages with the Euler-Maruyama method with a time step of $dt = 0.01$ ages, starting with 5 fixed cells initialized with 1 unit of G_1 and G_g. Cell growth was simulated using the same method as in the previous section. We generated a time course with 28 time points by sampling all cells from the simulation every 5 ages starting at age 15 (the chronological age at which the system had reached a near equilibrium state). We then induced dropouts in the data following BoolODE's procedure. Namely, genes with expression below the median expression level of all cells at a particular age were set to 0 with probability 0.5.

The data was then labeled by assigning cell types for each of the branches, $B1$, $B2$, and $B1+B2$, based on whether a cell exclusively expressed one of the terminal lineage markers G_{B12} or G_{B22} above a threshold of 1, or else expressed both above this same threshold. Lastly, cell fate probabilities were estimated by simulating 500 trajectories starting from each cell and computing the fraction of trajectories that ended in each of the three branches.

This simulation can be generated by downloading the BoolODE code from https://github.com/murali-group/BoolODE and running the script at https://github.com/ColeBoyle/global-StationaryOT/extra/data_preprocessing/sim/GRN_simulation. The exact BoolODE code we used is archived at https://doi.org/10.5281/zenodo.20723235.

To produce the results in Panels **D** and **E** of Figure 5, the time course was downsampled to $n_a = 25$ cells per age before applying gStatOT, StatOT, and PBA. All methods were given the true growth rates of the sampled cells, $R(X, a)$, and gStatOT and StatOT were given the integration

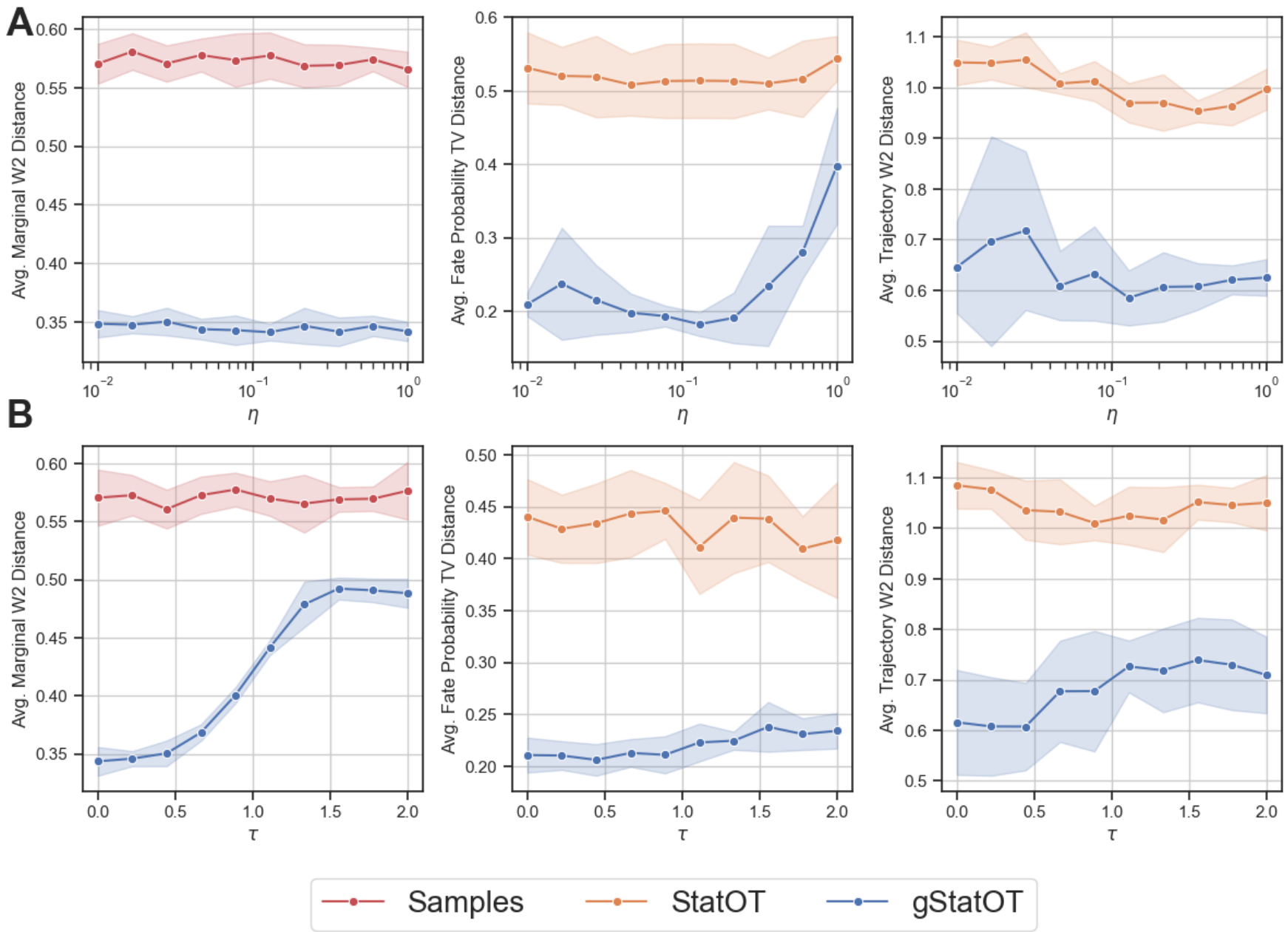

Figure SI.2: gStatOT's sensitivity to growth rate noise and misspecification.
We tested gStatOT's outputs on the simulated time course (with $T = 25$ samples of $n_a = 10$ cells) presented in Figures 3 and 4 of the main text. We used the same parameters as in the main text, but with noisy (Panel **A**) and misspecified (Panel **B**) growth rates. In Panel **A**, we gave gStatOT and StatOT growth rates subject to both additive and multiplicative noise at various levels. We used multiplicative noise to account for the large disparity in growth rates between cell states, and additive noise for the majority of cells that have a true growth rate of 0. Specifically, we set each cell's growth rate to $\hat{R}_i = R_i + \eta(R_i\mathcal{N}_1 + \mathcal{N}_2)$, where R_i is the true growth rate, $\mathcal{N}_1$ and $\mathcal{N}_2$ are independent standard normal random variables, and η is a noise level parameter selected to be 10 evenly spaced values in the logarithmic space between 0.01 and 1. We found gStatOT's performance to be relatively robust to this noise, with the fate probability error significantly deviating only once the noise level was on the same order of magnitude as the spatial noise $\sigma = \sqrt{0.1} \approx 0.31$. In Panel **B**, we again kept all parameters the same but gave both methods misspecified growth rates by giving an increasing number of uncommitted cells the 100 per day division rate of the fixed progenitor cells in our simulation. In particular, we defined these uncommitted cells to be ones with absolute Gene 2 expression less than 0.25, and Gene 1 expression less than τ, with τ selected to be 10 evenly spaced values between 0 and 1. Here, we found gStatOT's fate probability estimates to be stable, however, the marginal error steadily increased with τ, and a similar but smaller trend was observed in the trajectory error. This change in the marginal error is expected due to the significant imbalance in growth rates between the source and sink states. In the previous test, the noise was applied uniformly and so the relative difference in growth between source and sink states remained similar. In contrast, the large number of high growth cells forces the method to decrease the mass on these states in order to satisfy the equilibrium constraint, leading to erroneous marginals supported only on committed cell states. However, even with this misspecification, gStatOT's marginals were still seen as improvements over the raw samples. With both of the tests, we did not find significant trends in StatOT's performance, as its estimates were already quite poor and variable to begin with. Code for generating this figure can be found at https://github.com/ColeBoyle/global-StationaryOT/extra/.

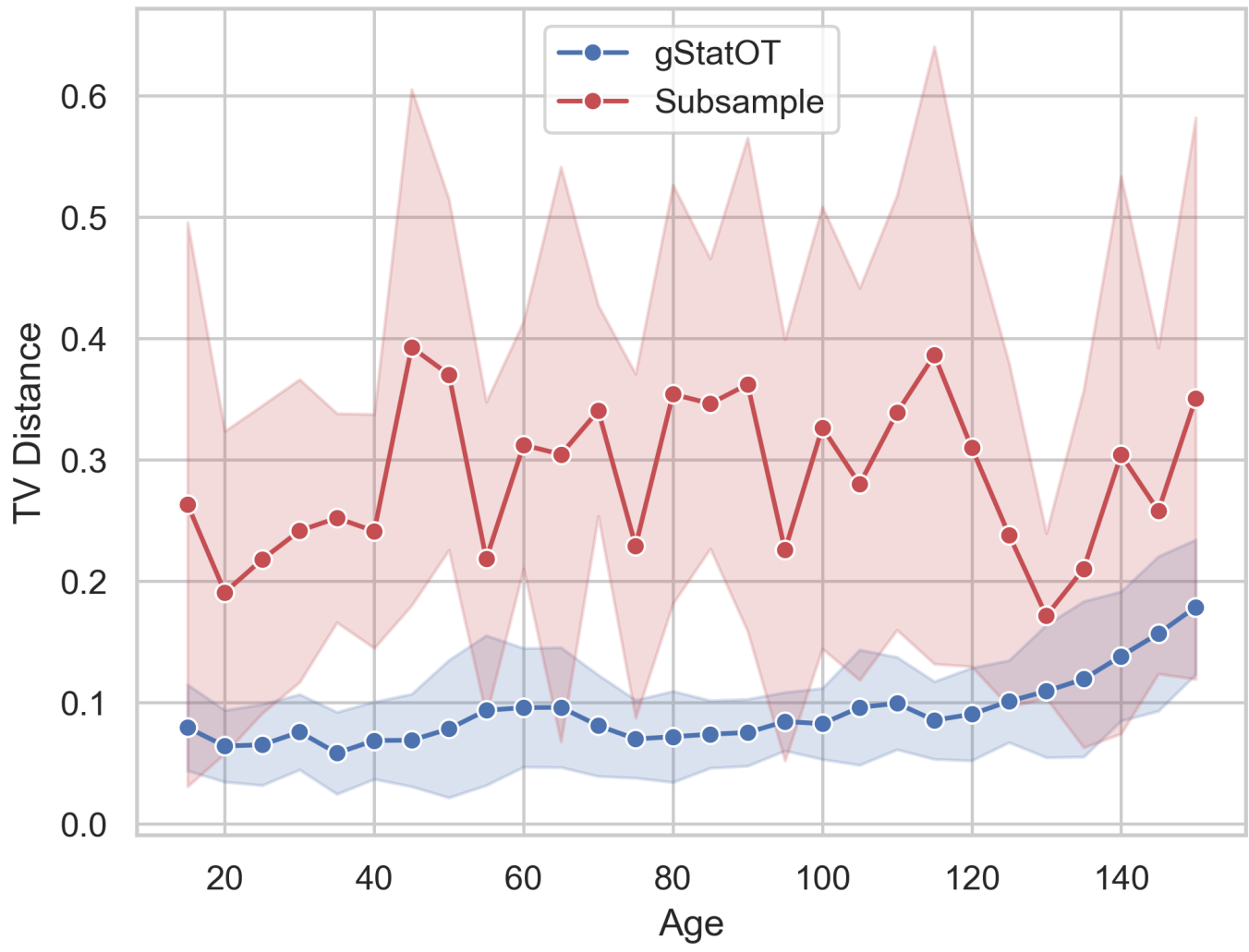

Figure SI.3: *The total variation distance between the proportions of cell death in the simulation and those estimated by gStatOT and the subsampled data (see (13)). The results are summarized by the mean and standard deviation of this distance across 10 reseeded simulations.*

time step dt as their Δt parameter. All other parameters were tuned to optimize for fate probability estimation, with the results and optimal choices shown in Section SI.7.2. With these parameters, the methods were run on new downsampled data from 10 reseeded simulations, and the results were summarized by taking the mean and standard deviation of each metric across the 10 runs.

To produce the results in Panel **F** of Figure 5, at every age we computed the total number of cells predicted to die in each branch over the time period dt, with each of the subsampled data, gStatOT, and the full simulated data. For gStatOT we used the parameters determined above. In particular, for all sink states, $\mathcal{X}^l_{\text{sink}}$, in each lineage, $l \in \{\text{B1}, \text{B2}, \text{B1+B2}\}$, we computed at every age, a,

$$m(l, a, \gamma_a) = \sum_{X \in \mathcal{X}^l_{\text{sink}}} (1 - e^{R(X,a)dt})\gamma_a(X) \tag{13}$$

where γ_a was taken to be the subsampled data, $\hat{\rho}_a$, gStatOT's reconstructed distribution, μ_a, and the full simulation samples, ρ_a. At each age we plotted the proportion of cell death in each lineage, $p_l(a, \gamma_a) = \frac{m(l,a,\gamma_a)}{\sum_{l'} m(l',a,\gamma_a)}$, for each of the three γ_a's. To quantify this comparison, we computed the total variation distance from the proportions estimated by the subsampled data and gStatOT to those estimated by the full simulation samples. The results are shown in Figure SI.3.

SI.5 Hematopoiesis preprocessing and parameter selection

We followed the preprocessing steps outlined in Li et al. [S14] to integrate their hematopoiesis data. Namely, 2000 variable genes were found on each log normalized sample independently, then Seurat's [S15] (V5.5) `IntegrateData` function was used to integrate the data after finding integration anchors using `FindIntegrationAnchors` with the default parameters. All integration and preprocessing scripts are available at https://github.com/ColeBoyle/global-StationaryOT/extra/data_

preprocessing/hematopoiesis. A 30-component PCA embedding was then computed on the integrated data. We then split the data into pre- and postnatal time courses as described in the text. The PCA embeddings of cells in each time course were then passed to both StatOT and PBA to establish ground truth estimates. We followed a similar procedure to that of Li et al. [S14] to fit the methods to the data at each age. In particular, we used the same sink states determined by Li et al. [S14] using marker genes for each of the 6 terminal lineages. For StatOT Δt was taken to be the default parameter 0.25, and the sink states were given a growth rate such that all mass is removed from these states within the period Δt. All other cells were given a small positive growth rate consistent with normalizing $\hat{g}_i$ after sink growth rates have been accounted for. We chose $\varepsilon = 0.04$ so that the total fate probability across all cells, for each of the 6 lineages, was roughly equal to the fraction of cells in the data that were labeled as being part of that lineage by Li et al. [S14] (see Figure SI.4). For PBA, we took $k = 20$ for its k-NN parameter and gave sink states flux rates, R_i, equal to $1/D$, where D is an estimated diffusion constant. All other cells were given a flux rate such that $\sum_i R_i = 0$. $D = 1$ was then selected the same way as ε for StatOT. As in [S14], we found that the methods produced fate probability estimates that were highly correlated with each other (see Figure SI.4). Separately for each method and age, we estimated the 80th percentile of the hitting time of sink states by sampling cell trajectories from the method's transition matrix. We then sampled cell trajectories of this length for use in the trajectory metric described in Section SI.6.

These methods were then reapplied to the downsampled data, as described in the text, and parameter sweeps were performed to select optimal parameters for each method, time course, sparsity level, and temporal resolution. gStatOT/StatOT were given the same growth rates and Δt value used when computing the StatOT ground truth on the full samples. PBA was given the same flux rates used when computing its ground truth on the full samples. As with the simulated data, we picked parameters using the average of our evaluation metrics, however since we are using StatOT as a proxy for the ground truth, when choosing gStatOT's parameters we weighted the marginal metrics twice as high as the others. SI.7.3.1 and SI.7.3.2 show the results of our parameter sweeps for variable sample sparsity and temporal resolution. The parameter values used in Figures 6 and 7 are highlighted in red. Note that for the $n_a = 500, 600$ and 700 samples sizes we took gStatOT's w parameter to be 10, whereas in all other experiments it was set to 1. See Figure SI.7 for the time it took to solve gStatOT for each sparsity level in Figure 6.

In Figure 7 **A**, fate probabilities were plotted on a UMAP of the entire integrated data set, with fading saturation to indicate the magnitude of each estimate. The points in gStatOT's plots were scaled by its reconstructed marginals, μ_i. Figure SI.5 and Figure SI.6 show similar plots with the inferred flow fields of gStatOT and StatOT at several prenatal and postnatal ages, respectively. These flow fields were computed by taking the row-normalized couplings, $\pi_i^\star$, and computing the average velocity of each cell state as $\sum_j (\pi_i^\star)_{kj}(X_j - X_k)$, where X_k is the UMAP embedding of the kth cell state. The flow fields were then plotted as streamlines using Matplotlib's [S16] `streamplot` function.

For Figure 7 **B**, we computed the Pearson correlation between the sparsely estimated fate probabilities of gStatOT/StatOT and those computed by StatOT for the same cells using the full data. The results were averaged across all ages and reported for each cell lineage.

See Section SI.6.4 for the details of how we computed and compared the lineage driver gene list of each method to produce Figure 7 **C**.

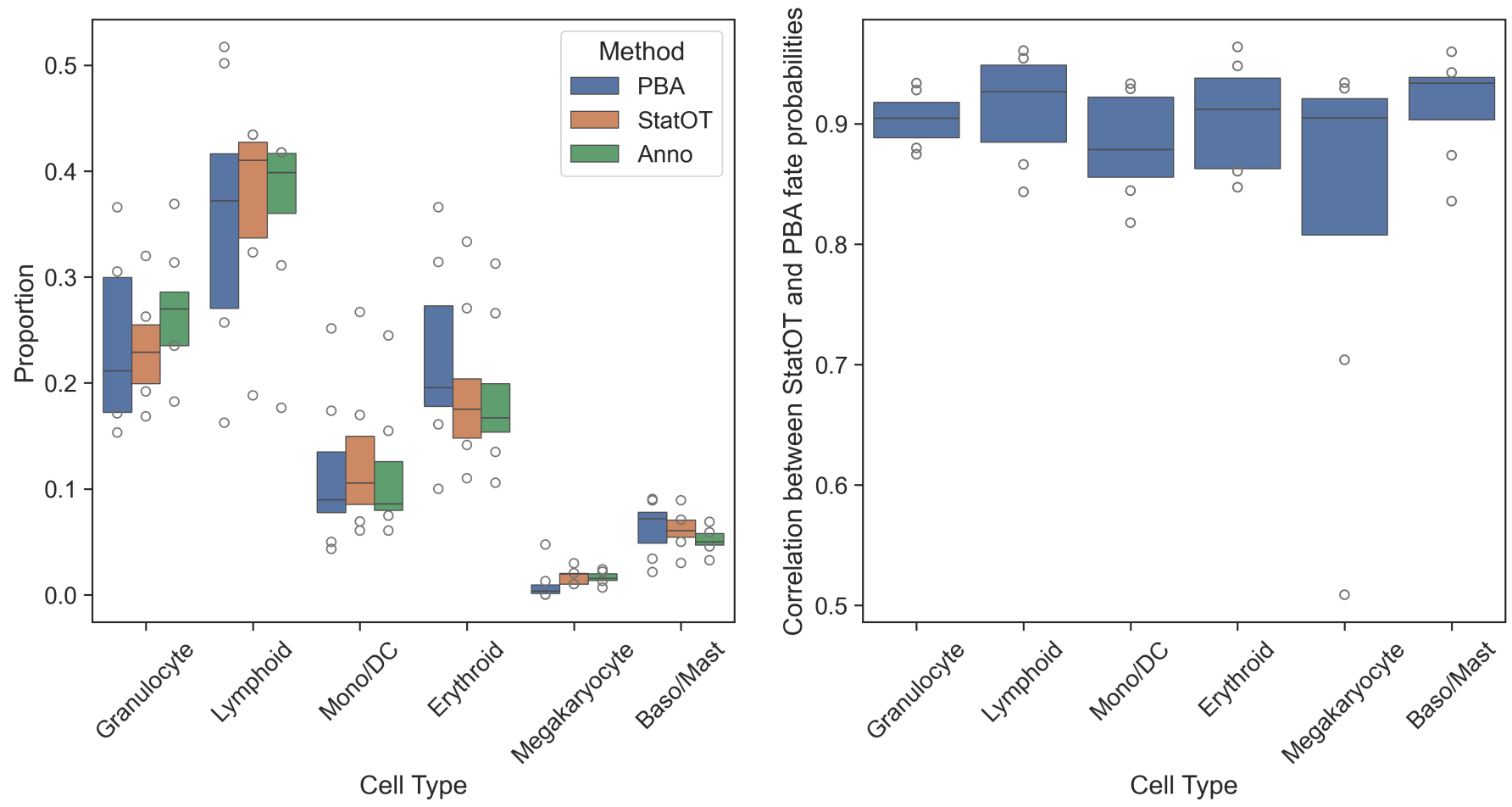

(a) Prenatal

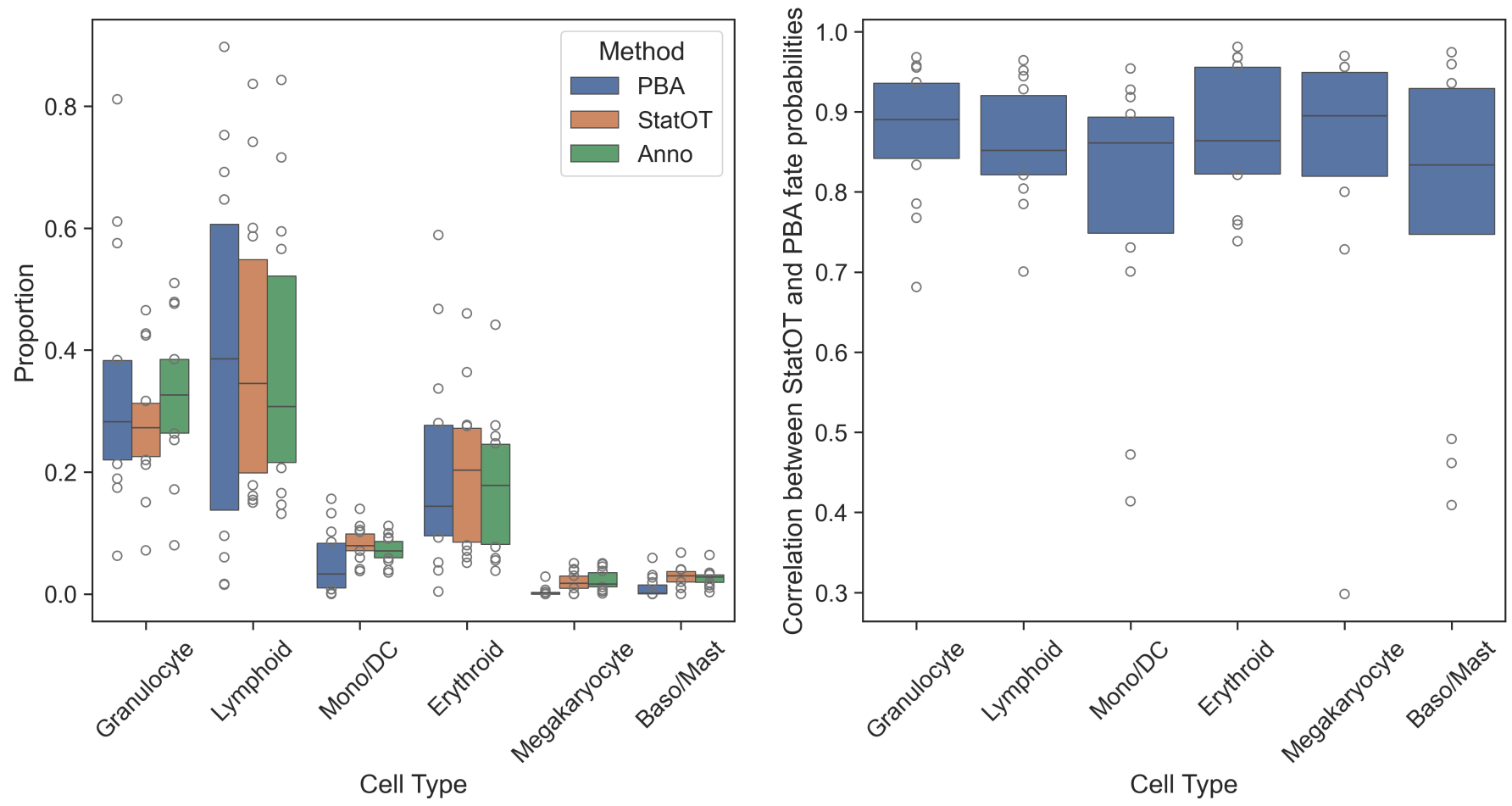

(b) Postnatal

Figure SI.4: Left: *Fate probability proportions, for each lineage estimated by StatOT and PBA on the full data, and the proportion of cells in the data annotated as being part of each lineage. Each proportion is the sum of all the cells' probability of ending in that lineage. StatOT's ε and PBA's D parameters were chosen so that each fate probability proportion roughly matched the annotated proportions.* ***Right:*** *The Pearson correlation between the fate probability estimates of StatOT and PBA on the full data.*

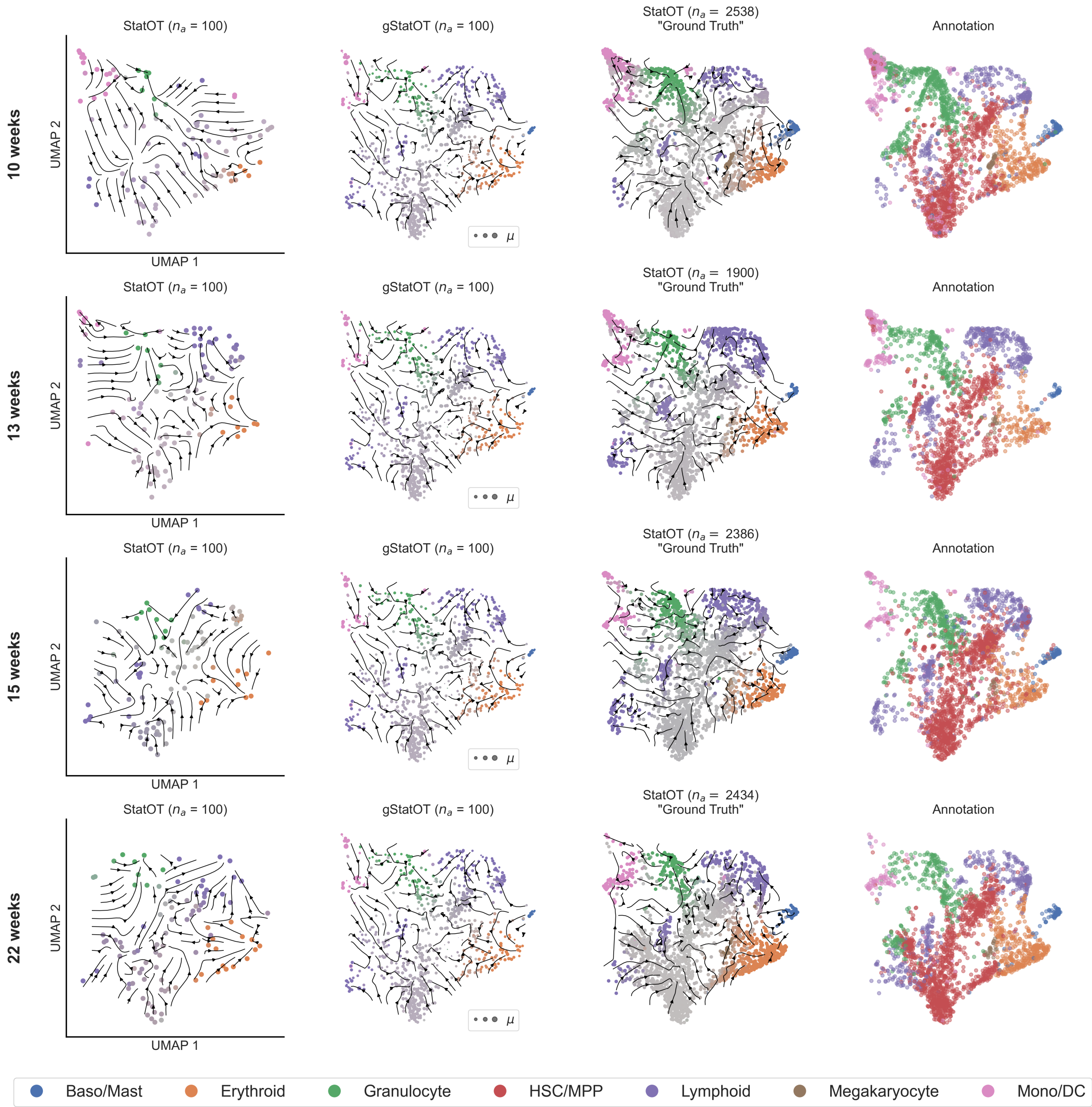

Figure SI.5: *Same as Figure 7* ***A****, but with the inferred flow fields and fate probabilities of gStatOT and StatOT at several prenatal ages.*

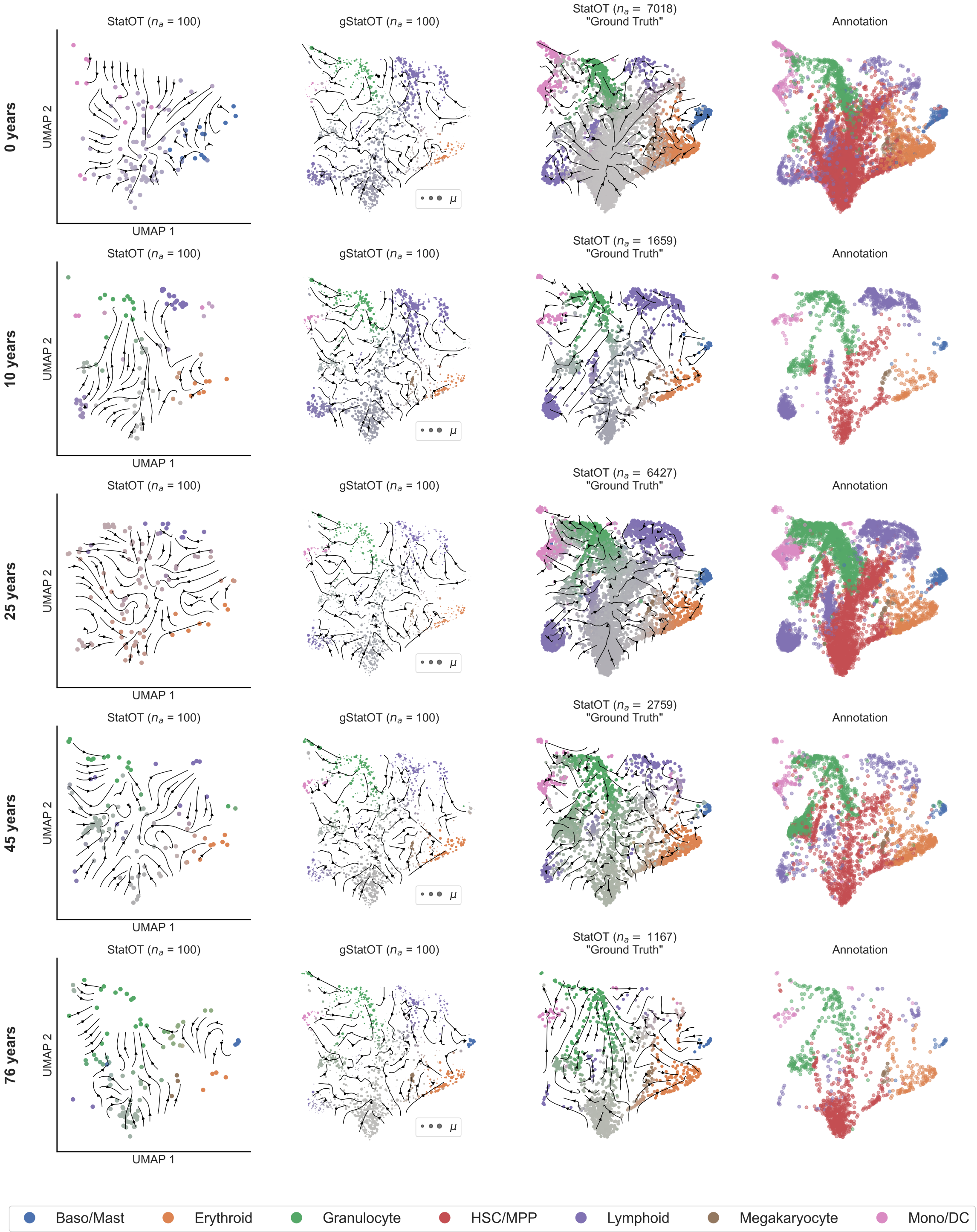

Figure SI.6: *Same as Figure 7* ***A****, but with the inferred flow fields and fate probabilities of gStatOT and StatOT at several postnatal ages.*

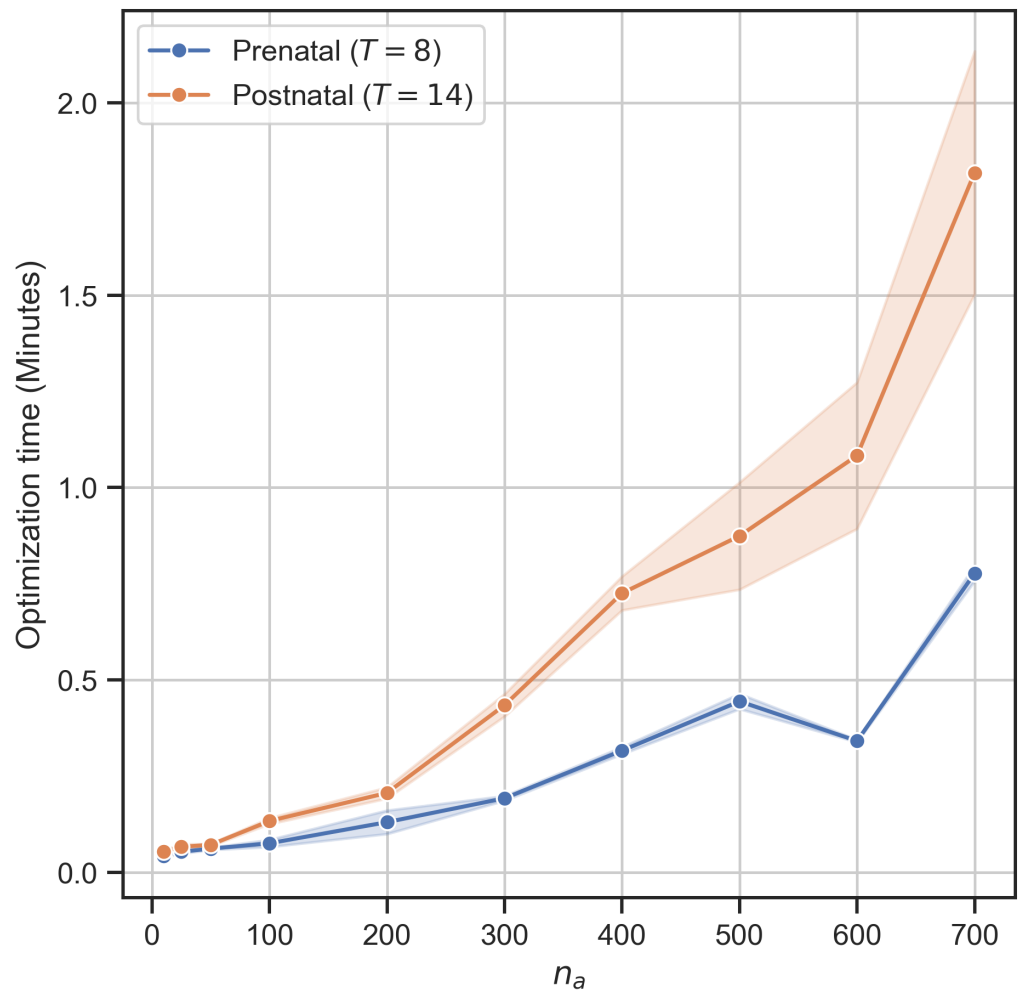

Figure SI.7: *The time it took to solve gStatOT to produce the results in Figure 6 Panel* ***A*** *for each sparsity level, n_a. The time was averaged over the 10 validation runs. Each run was performed on a single NVIDIA H100 80GB HBM3 MIG instance (profile: 3g.40gb).*

SI.6 Evaluation Metrics

SI.6.1 Marginal Error

The second marginal of each optimal $\pi_i^\star$ recovered by gStatOT provides an estimate of the population distribution at age a_i. In order to test its debiasing ability, before applying the method to the simulated and real data, we downsample our initial samples and then compute the $\mathbb{W}_2$ distance between each $\mu_i = (\pi_i^\star)^\top \mathbb{1}_N$ and the full sample of data, which we denote ρ_{a_i}. That is, we report

$$\mathbb{W}_2(\mu_i, \rho_i) = \left(\min_{P \in \Pi(\mu_i, \rho_i)} \langle C, P \rangle \right)^{1/2} \tag{14}$$

where $C_{kl} = \|X_k - Y_l\|^2, X_k \in \text{supp } \mu_i, Y_l \in \text{supp } \rho_i$. In Figure 4 panel **B** and Figure 6 panels **B**, **C**, we plot $\frac{1}{T} \sum_i^T \mathbb{W}_2(\mu_i, \rho_i)$ for each value of T.

SI.6.2 Trajectory Metric

The Markov transition matrix P_i obtained from either StatOT, gStatOT, or PBA provides an estimate of the cell dynamics at age a_i. We obtain a distribution, $\hat{\mathcal{T}}_i$, of cell trajectories at each age by sampling, $N_{\mathcal{T}_i}$, paths from P_i over a time τ starting from cells with positive growth. Letting $r_{ij} = \max\{\hat{R}_{ij}, 0\}$, the initial cells at age a_i are sampled from the discrete distribution $(r_i / \sum_j r_{ij}) \odot (\pi_i^\star)^\top \mathbb{1}_N$. We report the $\mathbb{W}_2$ cost between $\hat{\mathcal{T}}_i$ and the ground truth $\mathcal{T}_i$, which we describe obtaining in the text, with the cost

$$C_{lm} = \frac{1}{\lfloor \tau / \Delta t \rfloor} \sum_{k=0}^{\lfloor \tau / \Delta t \rfloor - 1} \|f_l(k\Delta t) - g_m(k\Delta t)\|^2, f_l \in \text{supp } \hat{\mathcal{T}}_i, g_m \in \text{supp } \mathcal{T}_i \tag{15}$$

SI.6.3 Fate probability metric

From each P_i we computed each cell's lineage fate probabilities across K lineages, $\hat{B}_i := \hat{B}_{a_i} \in \mathbb{R}^{N\times K}$, as in Section SI.3 for gStatOT/StatOT and used PBA's built-in method to obtain its estimates. For each sampled cell, $X_j \in \hat{\rho}_i$, in the downsampled time series, their predicted lineage fate probability distribution, $(\hat{B}_i)_{j\cdot}$, was compared to ground truth probabilities $(B_i)_{j\cdot}$ using the total variation distance

$$\mathrm{TV}((\hat{B}_i)_{j\cdot}, (B_i)_{j\cdot}) = \frac{1}{2}\sum_{k=1}^{K} |((\hat{B}_i)_{jk} - (B_i)_{jk})| \tag{16}$$

We report the average TV distance over all sampled cells at each age, $\frac{1}{n_i}\sum_{j=1}^{n_i} \mathrm{TV}((\hat{B}_i)_{j\cdot}, (B_i)_{j\cdot})$ in Figures 4 and 5. In plots where we vary the number of time points, we report the average of this metric across all ages. As noted above, if a method failed to compute fate probabilities at a certain age, we set the total variation metric to its maximum value of 1 for that age.

SI.6.4 Driver gene identification and average precision scoring

As done by Weiler et al. [S11], we use the correlation between gene expression and cell fate probability to produce ranked lists of putative driver genes at each age in the hematopoiesis time course. Before computing these correlations, we log-normalized the count data using the `normalize_total` (with `target_sum=`10^4) and `log1p` functions from the `scanpy` Python package [S17].

Since gStatOT's marginals are supported over all sampled data, we use a weighted correlation. Letting G_l be the column corresponding to a gene l in the $N \times L$ (log-normalized) gene expression matrix, we compute for weights $w \in \mathbb{R}^N$ such that $\sum_j w_j = 1$

$$\mathfrak{m}_w(G_l) = \sum_{j}^{N} w_j (G_l)_j, \quad \mathfrak{m}_w((B_i)_{\cdot k}) = \sum_{j}^{N} w_j (B_i)_{jk} \tag{17}$$

$$\mathrm{Cov}_w(G_l, (B_i)_{\cdot k}) = \sum_{j}^{N} w_j ((G_l)_j - \mathfrak{m}_w(G_l))((B_i)_{jk} - \mathfrak{m}_w((B_i)_{\cdot k})) \tag{18}$$

$$\mathrm{Corr}_w(G_l, (B_i)_{\cdot k}) = \frac{\mathrm{Cov}_w(G_l, (B_i)_{\cdot k})}{\sqrt{\mathrm{Cov}_w(G_l, G_l)\mathrm{Cov}_w((B_i)_{\cdot k}, (B_i)_{\cdot k})}} \tag{19}$$

Recalling that $\mathcal{X}^{a_i}$ denotes the set of cells sampled at age a_i, for StatOT we use the weights $w_j = \frac{1}{n_i}$ if cell $X_j \in \mathcal{X}^{a_i}$ otherwise $w_j = 0$, while for gStatOT $w_j = (\mu_i)_j$.

At each age, a_i, and for each of the cell lineages we use our ground-truth estimates to form ranked lists, $H^{m,a_i,k} = [\mathcal{G}_{l_1}, ..., \mathcal{G}_{l_m}]$, containing the top $m = 10$ genes most correlated with the kth lineage. To evaluate a method's ability to reconstruct these lists from downsampled data, we calculated the average precision (AP) [S18, S19] of each method's list and reported the mean AP (mAP) over all ages in Figure 7 Panel **C**. In particular, given an estimated ranked list of driver genes for lineage k, $\hat{H}^{a_i,k}$, we evaluated

$$\mathrm{mAP}(m, k) = \frac{1}{T}\sum_{i=1}^{T} \mathrm{AP}(\hat{H}^{a_i,k}; H^{m,k,a_i}) \tag{20}$$

where for an estimated and ground truth lists H and $\hat{H}$, respectively,

$$\mathrm{AP}(\hat{H}; H) = \frac{1}{|H|} \sum_{l=1}^{L_{\max}} \mathrm{prec}(\hat{H}[:l]; H) \mathrm{ind}(\hat{H}[l]; H) \tag{21}$$

$$\mathrm{prec}(\hat{H}; H) = \frac{|H \cap \hat{H}|}{|\hat{H}|} = \frac{\text{True Positives}}{\text{True Positives} + \text{False Positives}} \tag{22}$$

$$\mathrm{ind}(\mathcal{G}_l; H) = 1 \text{ if } \mathcal{G}_l \in H, 0 \text{ otherwise} \tag{23}$$

Here $H[l]$ is the lth element of H, and $H[:l]$ denotes the first l items of the list. We set $L_{\max} = 1000$, since relevant genes found outside this range will not significantly contribute to the average precision.

To provide a baseline measure of the ground-truth lists' recoverability, we evaluated the temporal consistency (GT-TC) using the AP between the ground-truth lists at adjacent ages. Precisely,

$$\begin{aligned}\mathrm{mAP}^{\text{GT-TC}}(m, k) = \frac{1}{T} \Bigg[& \mathrm{AP}(H^{m,k,a_2}; H^{m,k,a_1}) + \mathrm{AP}(H^{m,k,a_{T-1}}; H^{m,k,a_T}) \\ & + \frac{1}{2} \sum_{i=2}^{T-1} [\mathrm{AP}(H^{m,k,a_{i+1}}; H^{m,k,a_i}) + \mathrm{AP}(H^{m,k,a_{i-1}}; H^{m,k,a_i})] \Bigg]\end{aligned}$$

To compare with a simpler method, on the downsampled data, we obtained a ranked list of the top differentially expressed genes for each cell type relative to the rest of the data at each age using Scanpy's [S17] `rank_genes_groups` function. We then computed the mAP using Equation 20.

SI.7 Parameter sweeps

SI.7.1 Synthetic data: Aging epigenetic landscape

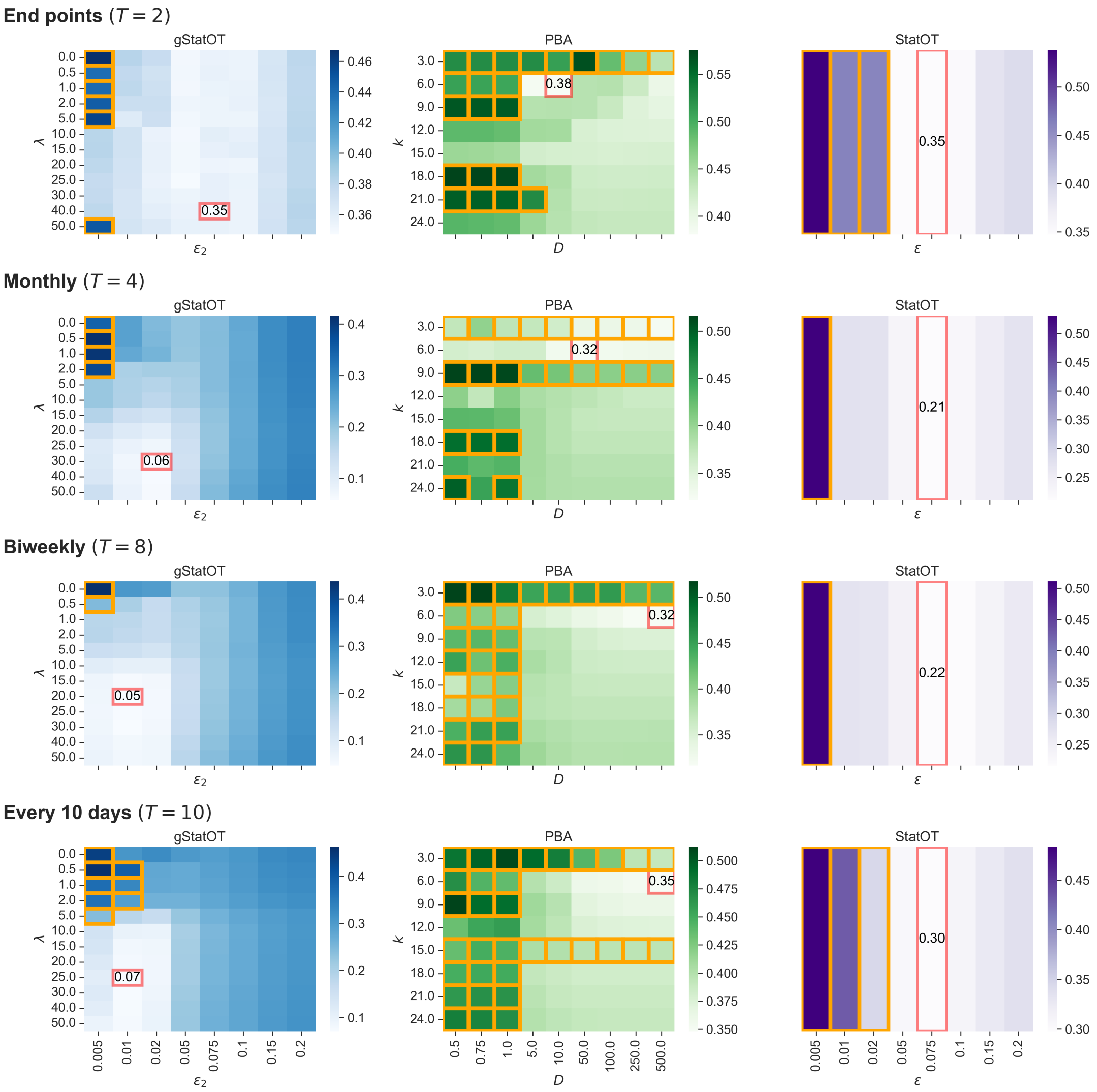

Figure SI.8: Average performance over the marginal, trajectory and fate probability metrics for parameter sweeps of gStatOT, StatOT, and PBA on our initial synthetic simulation. Parameters used for the results depicted in Figures 3 and 4 in the main text are highlighted in red. Parameter combinations that failed to compute fate probabilities at any age are highlighted in orange. Metrics were scaled by their minimum and maximum values over all method and parameter choices before taking the mean. Colour gradients are normalized for each method and value of T.

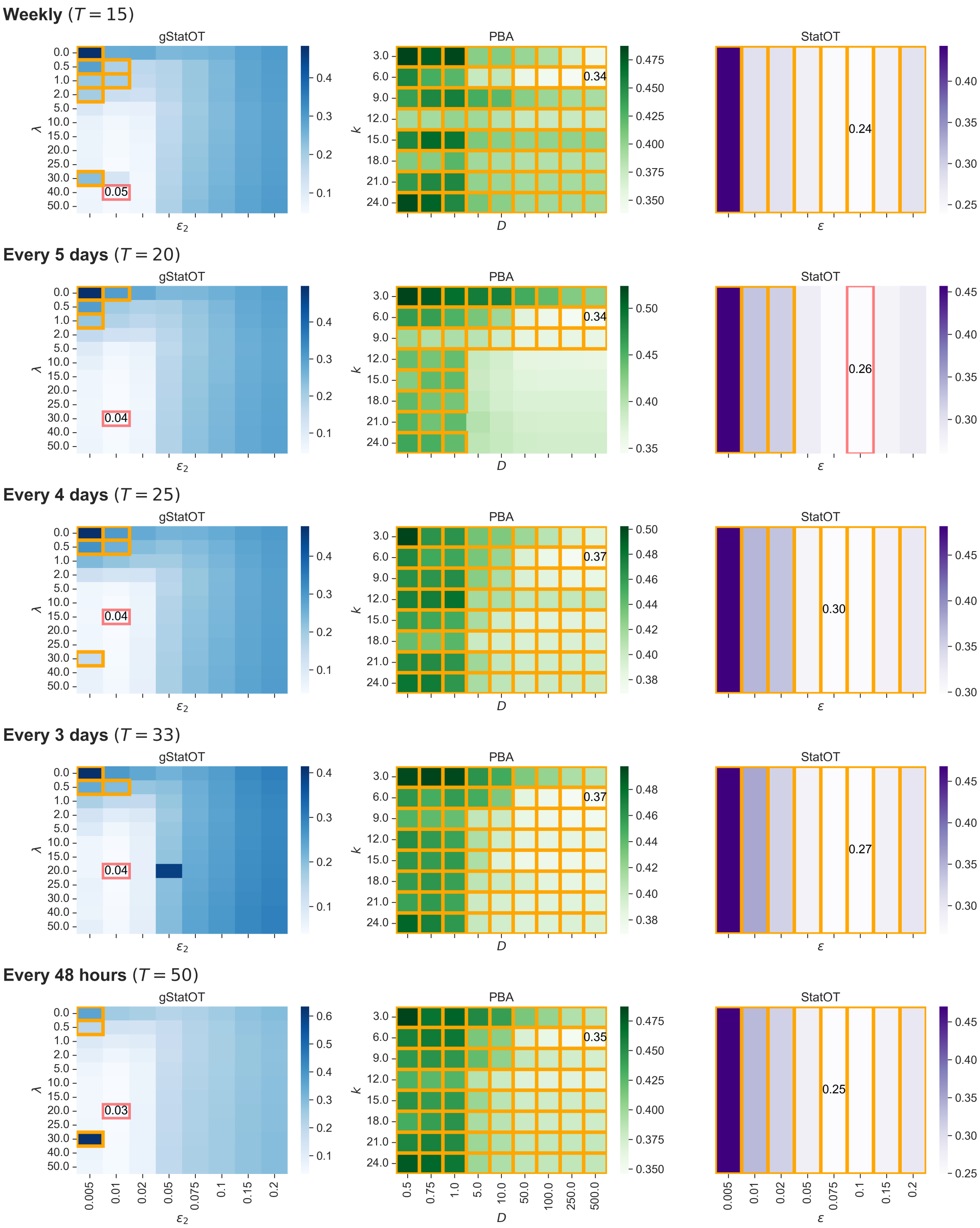

Figure SI.9: *Continued from [Figure SI.8](#), but for larger values of T.*

SI.7.2 Synthetic data: Loss of cellular identity over age induced by epigenetic erosion

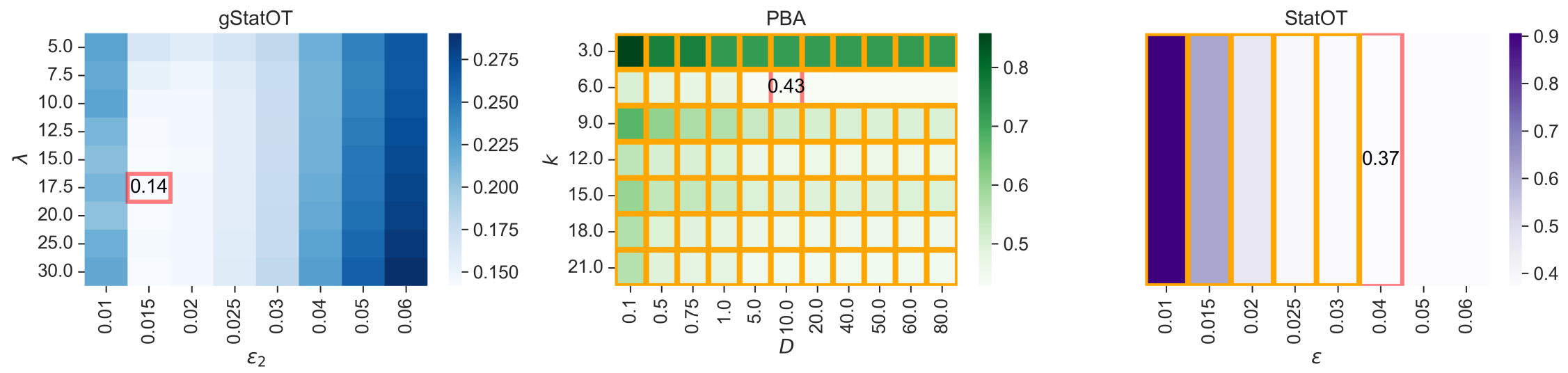

__Figure SI.10:__ Fate probability TV error for parameter sweeps of gStatOT and StatOT on the simulated GRN data. As in the previous figures, the parameters used for the results depicted in Figure 5 in the main text are highlighted in red. Colour gradient is normalized over each method's results.

SI.7.3 Hematopoiesis

SI.7.3.1 Variable Sample Size

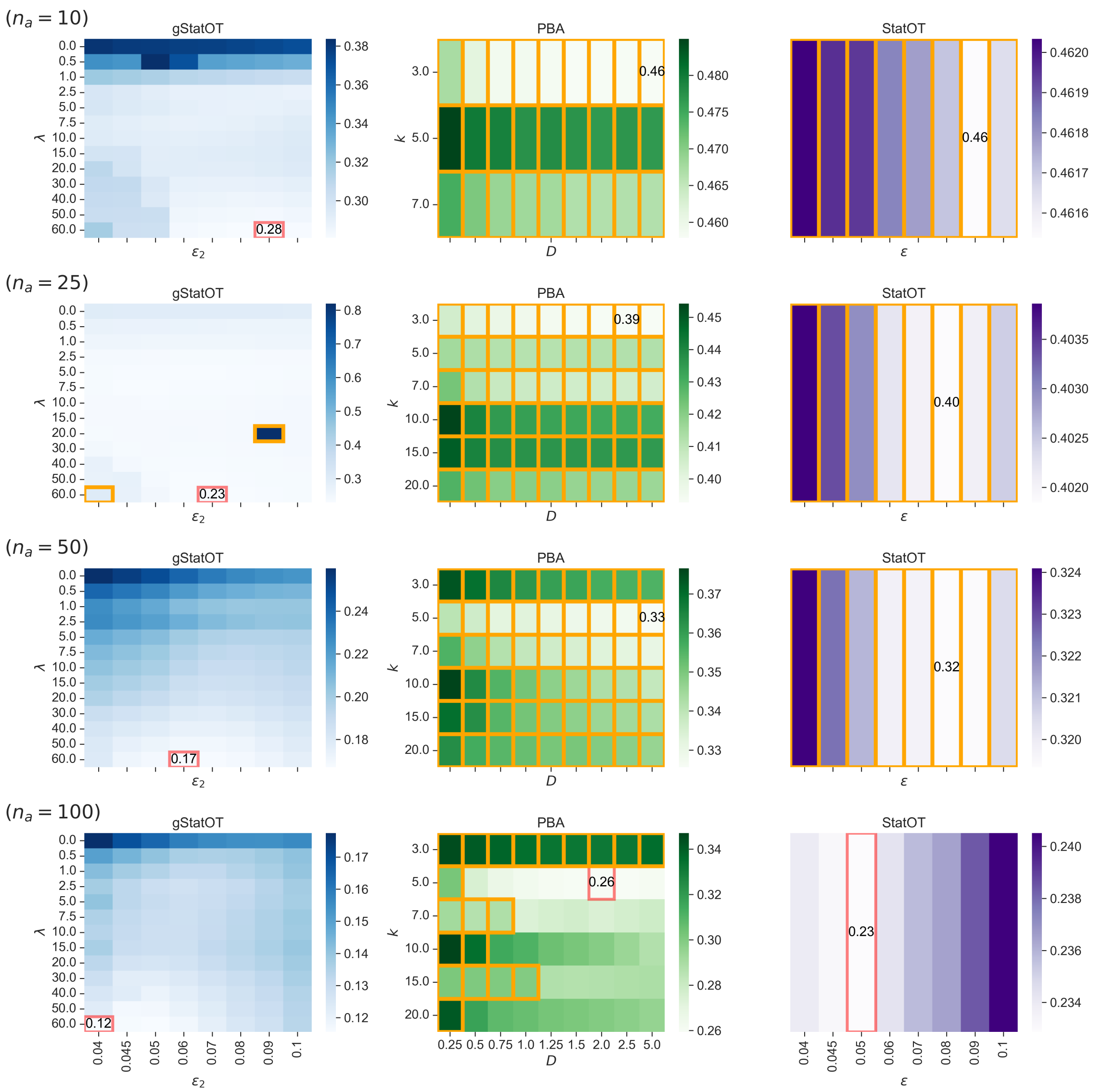

Figure SI.11: *As in* [*Figure SI.8*]*, but for parameter sweeps of gStatOT, StatOT, and PBA on the down-sampled prenatal hematopoiesis data from Li et al. [S14], at varying sparsity levels. Metrics were scaled by their minimum and maximum values over all method and parameter choices before taking the weighted mean, valuing marginal reconstruction twice as much as the other metrics. The parameters used for the results displayed in Figures 6 and 7 in the text are highlighted in red. The colour gradient is normalized over gStatOT's results for each value of n.*

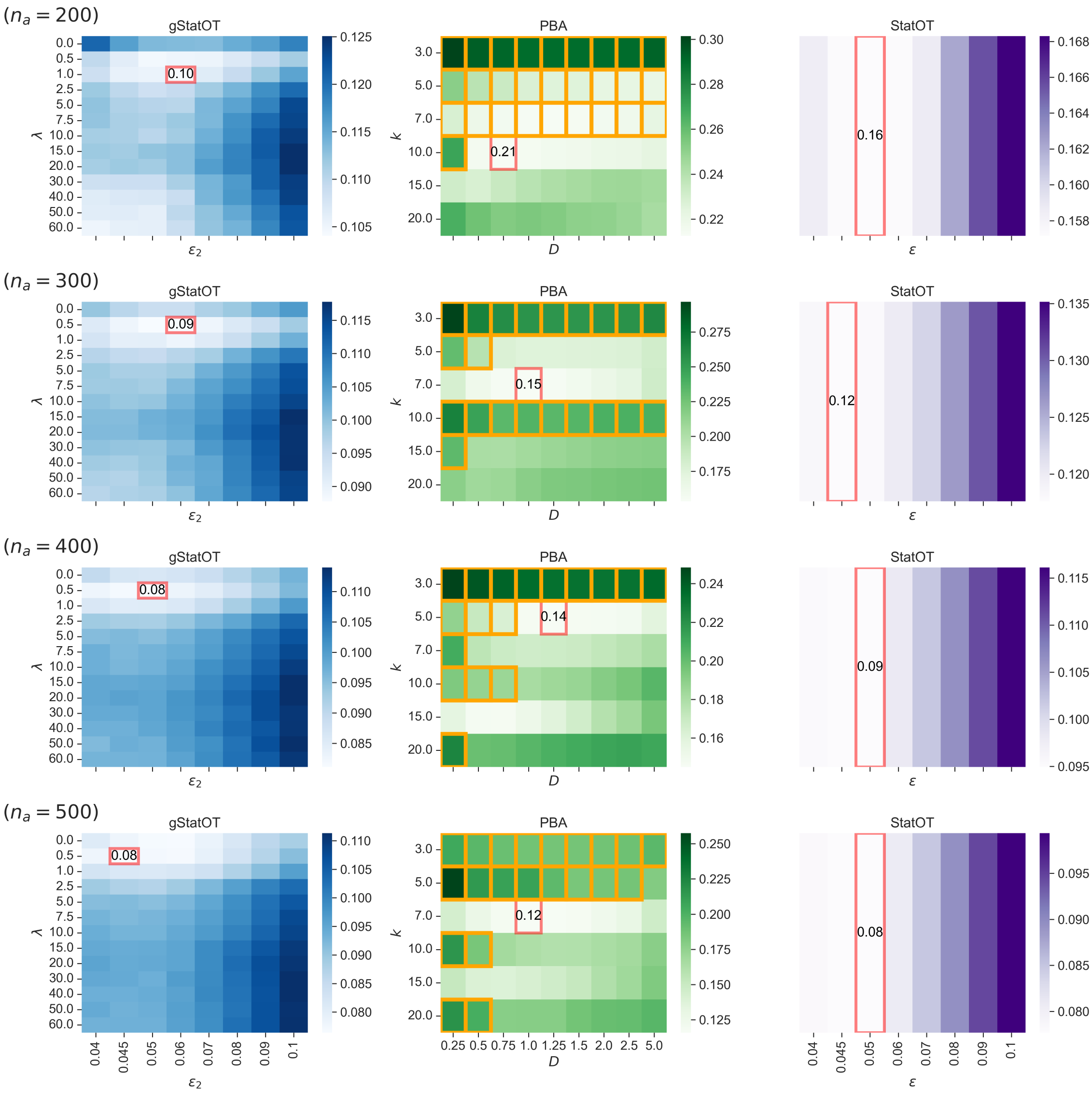

***Figure SI.12:** As in Figure SI.11, but for larger sample sizes.*

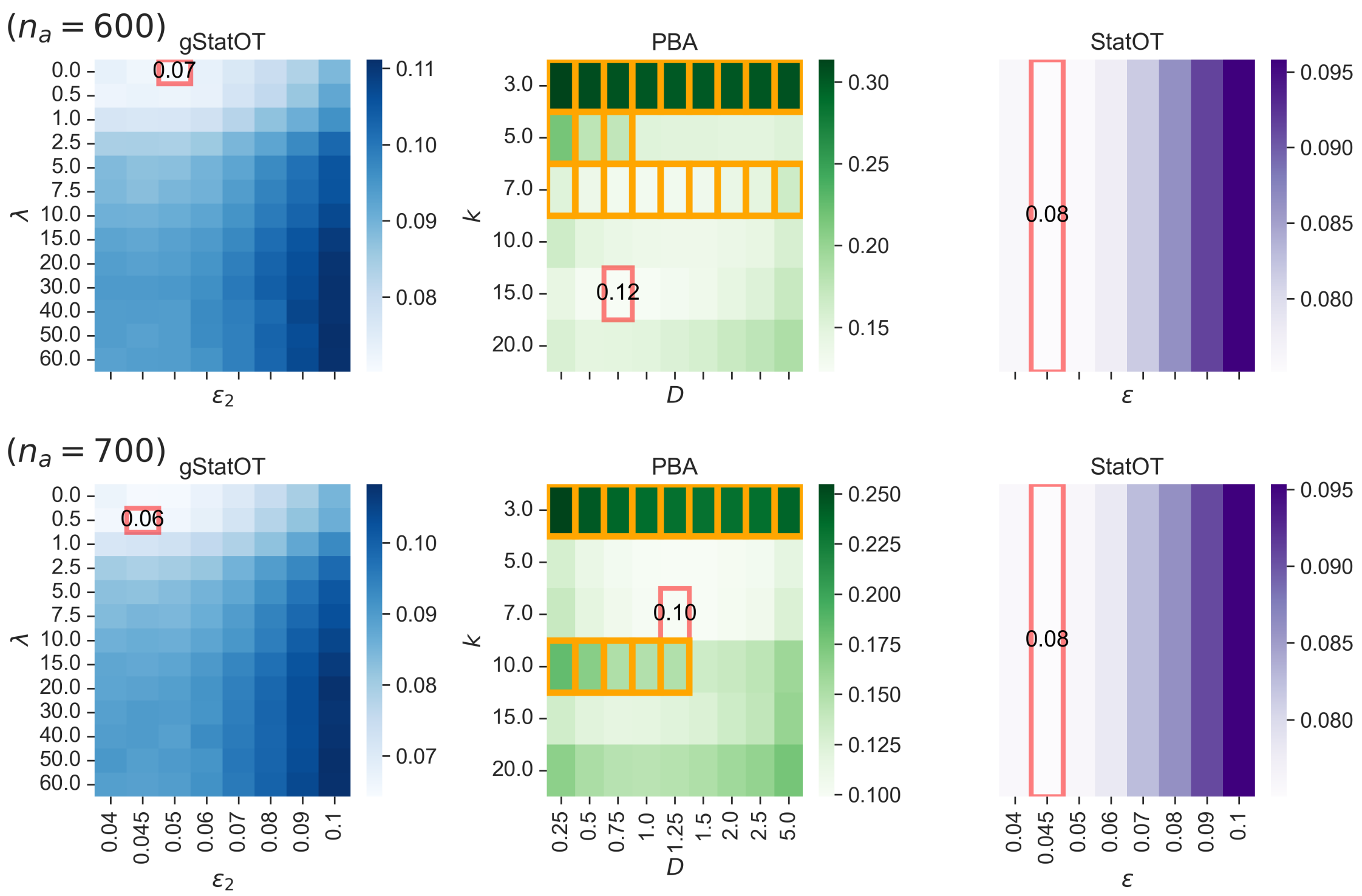

Figure SI.13: *As in Figure SI.11, but for larger sample sizes.*

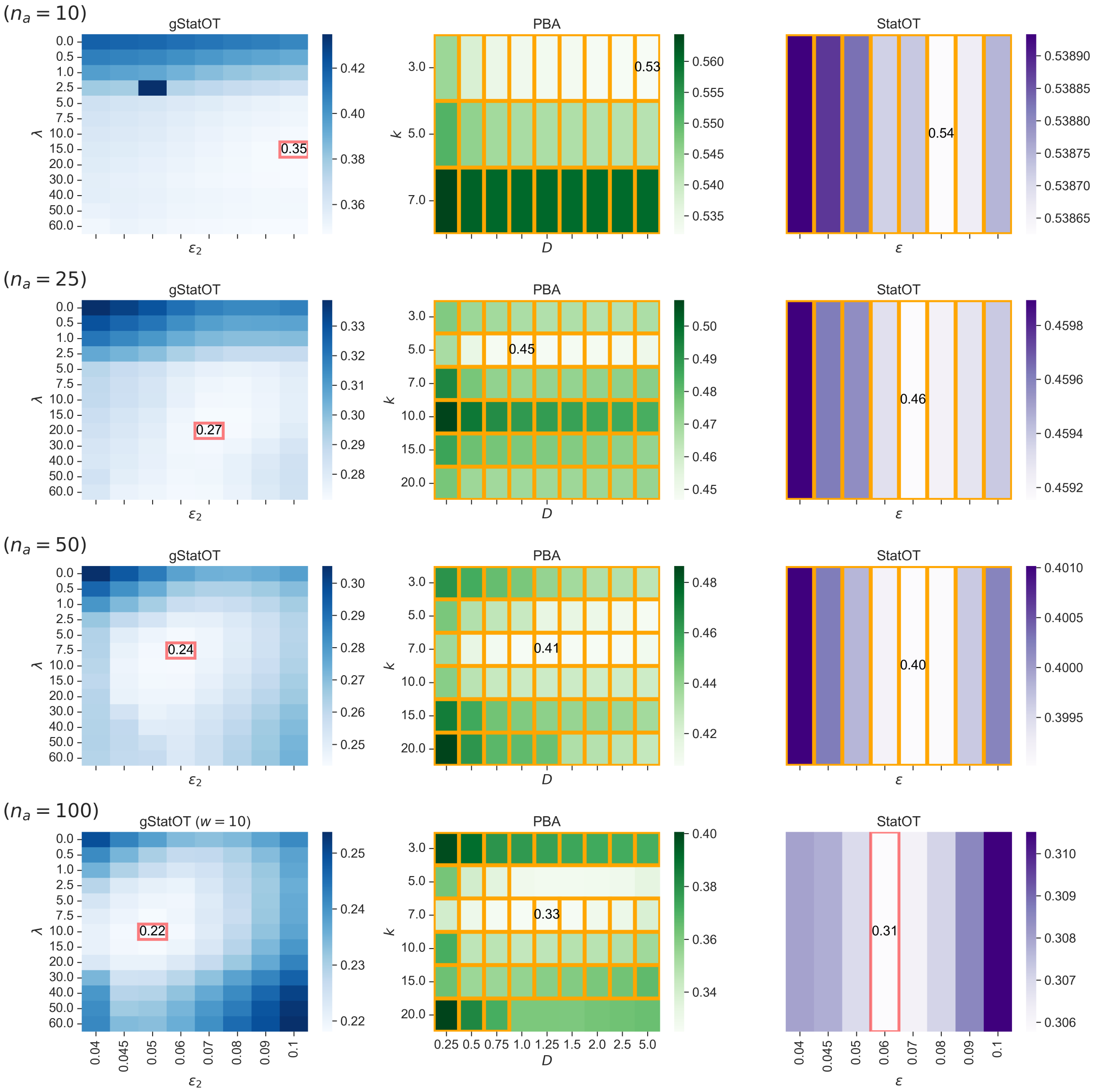

Figure SI.14: *As in Figure SI.11, but for the postnatal time course.*

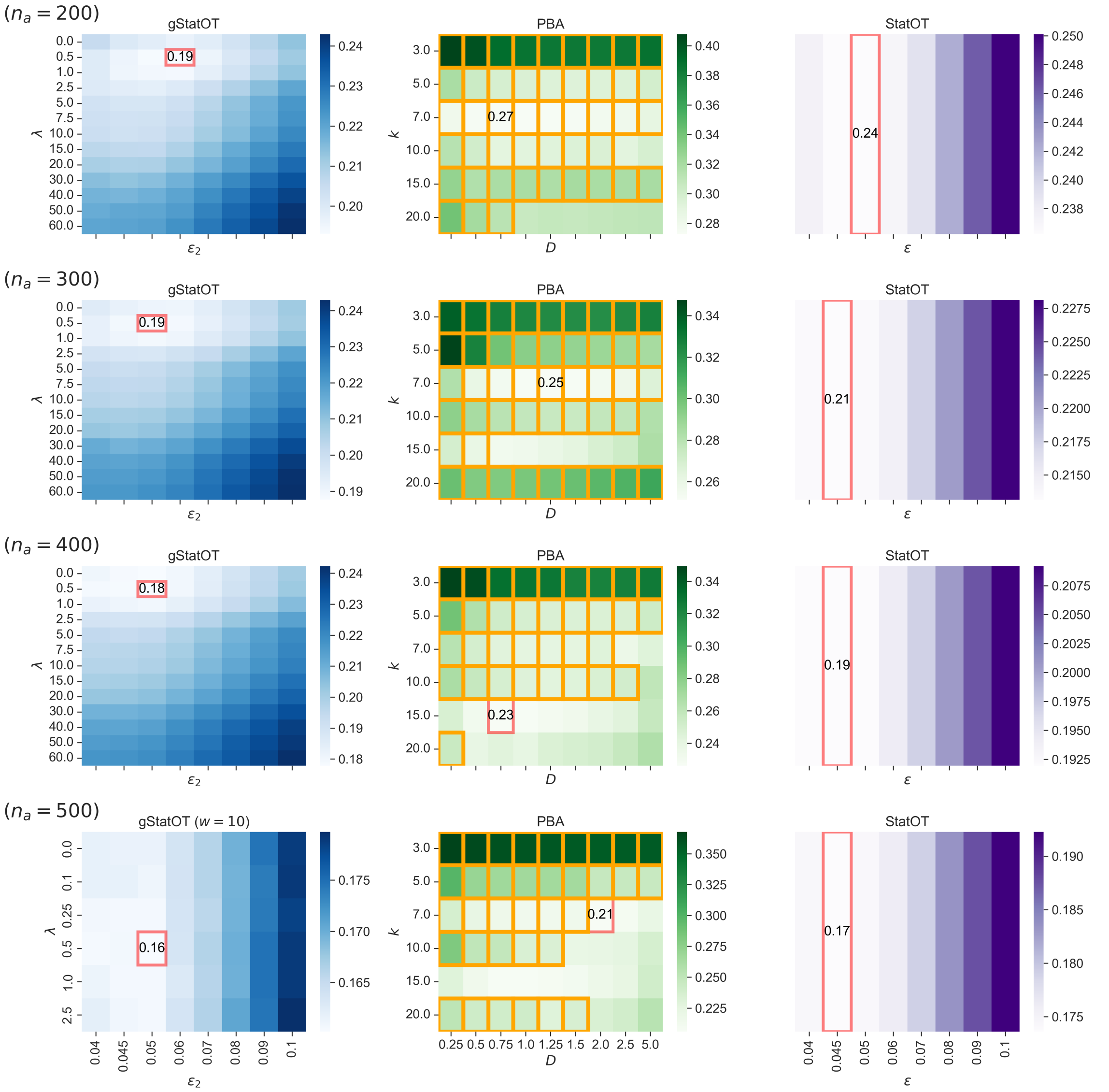

Figure SI.15: *As in Figure SI.14, but for larger sample sizes. Note we have taken $w = 10$ when fitting gStatOT at $n_a = 500$ sparsity levels.*

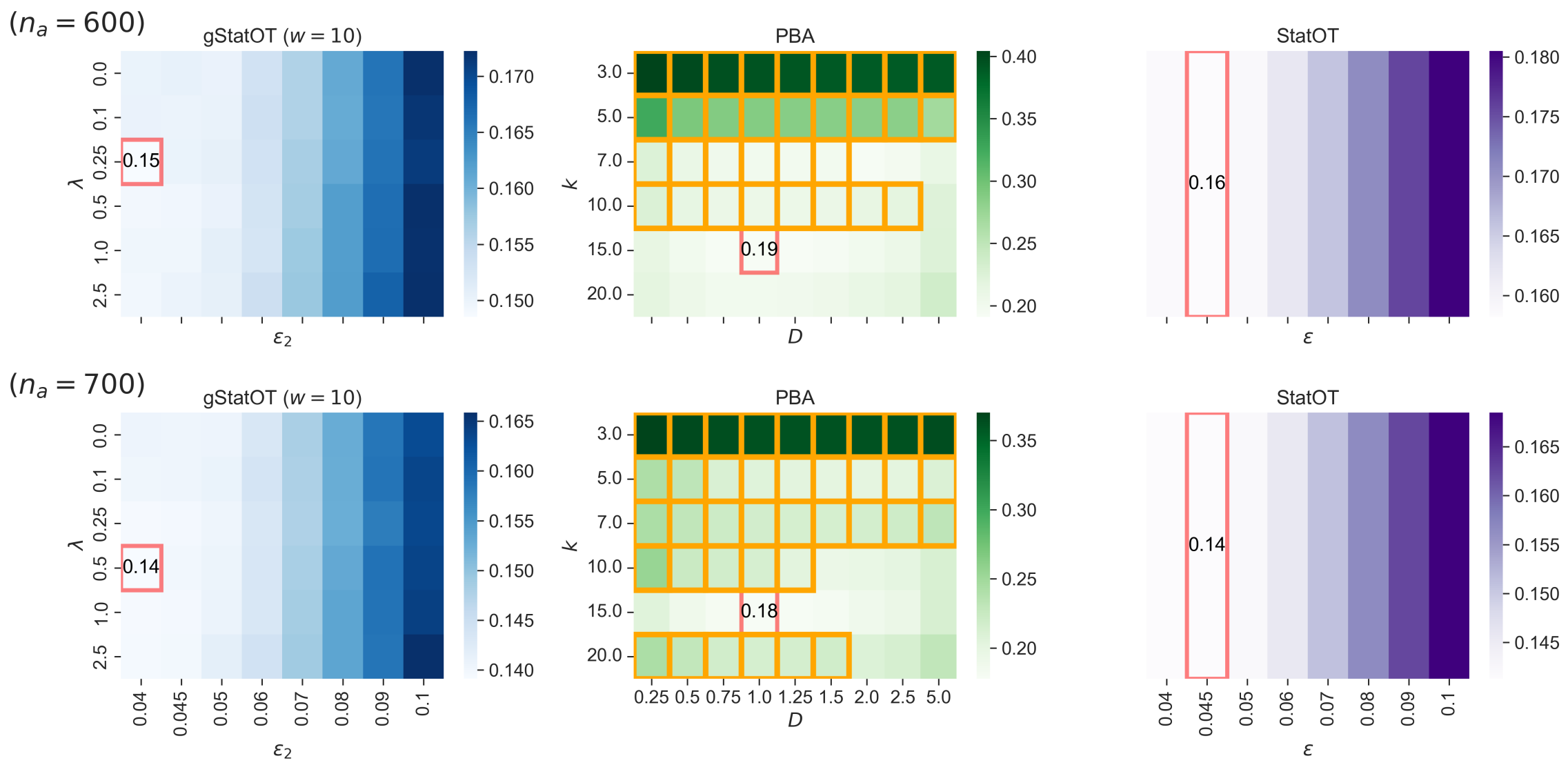

__Figure SI.16:__ As in Figure SI.15, but for larger sample sizes. Note we have taken $w = 10$ when fitting gStatOT at these sparsity levels.

SI.7.3.2 Variable Number of Time Points

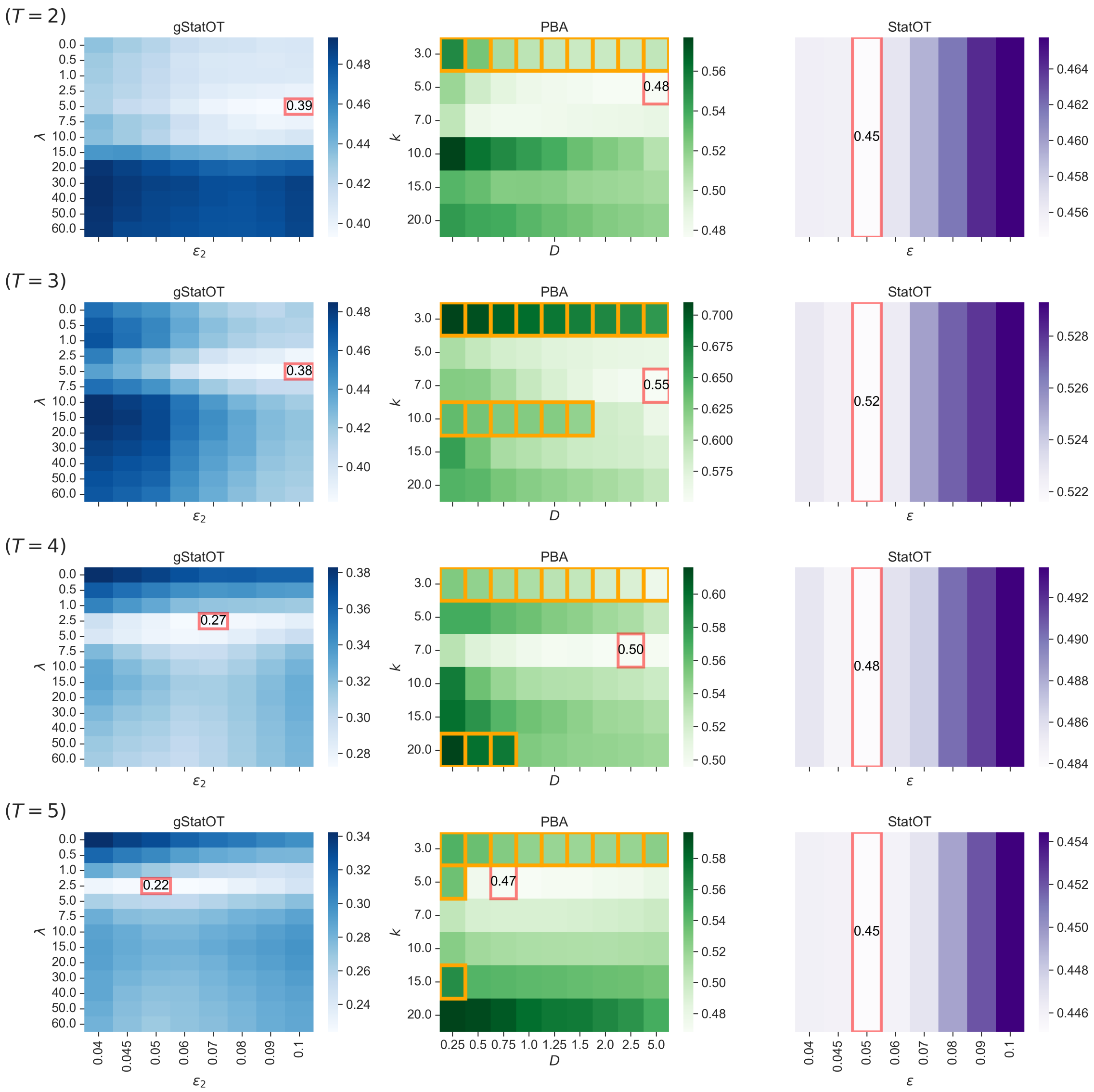

Figure SI.17: *As in Figure SI.11, but for the prenatal time course with varying number of time points.*

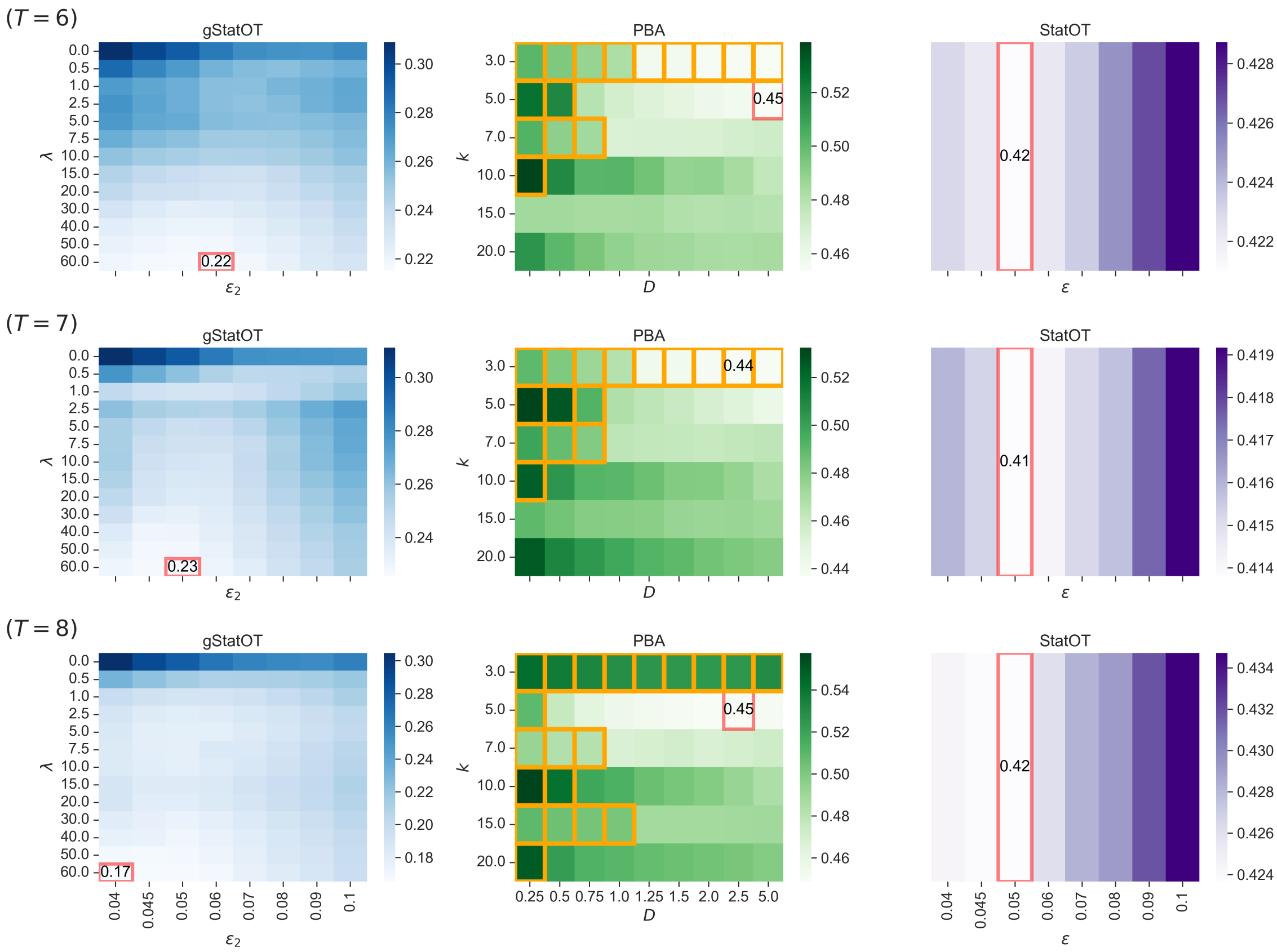

Figure SI.18: *Continuation of Figure SI.17.*

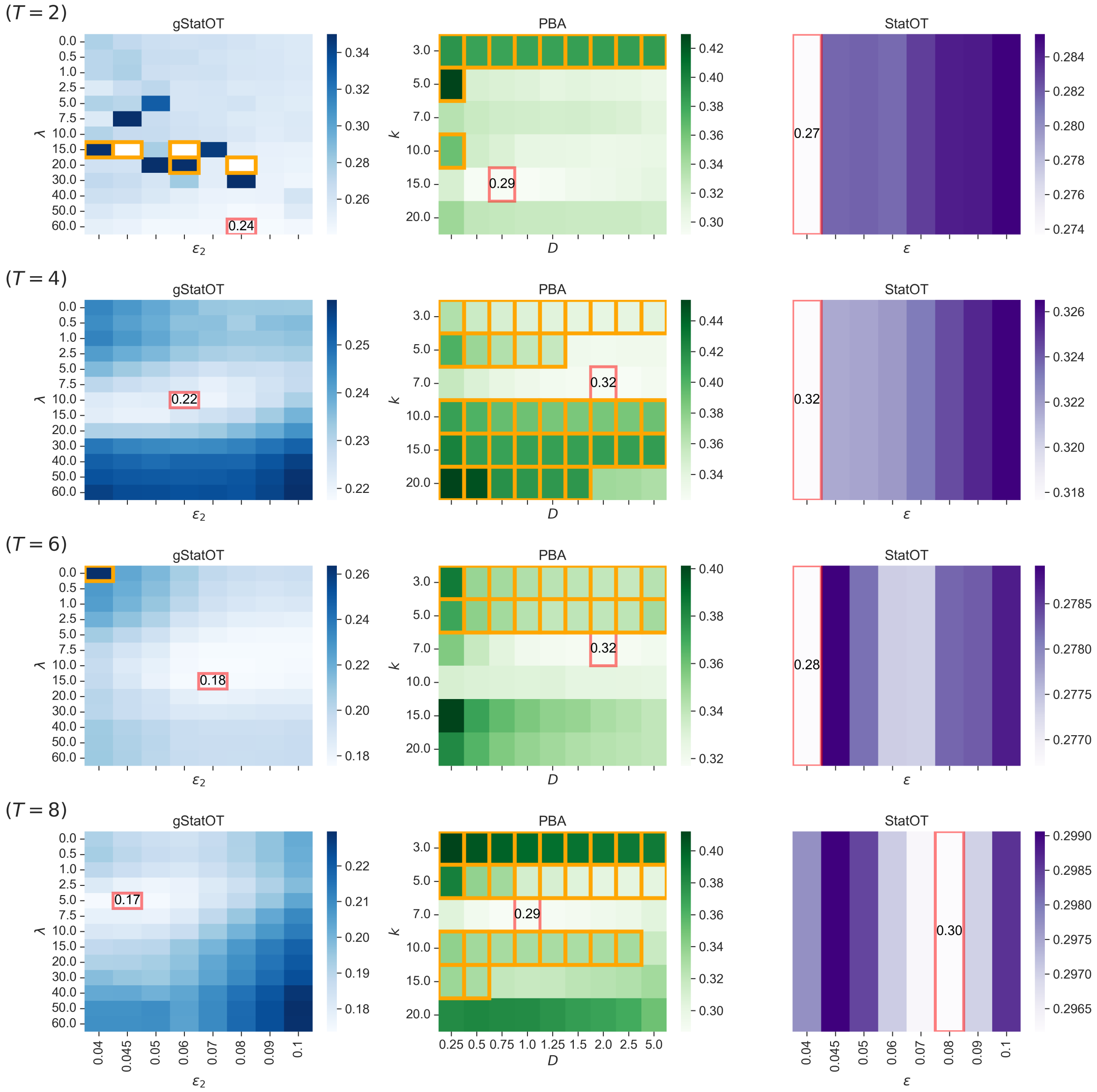

Figure SI.19: *As in Figure SI.17, but for the postnatal time course.*

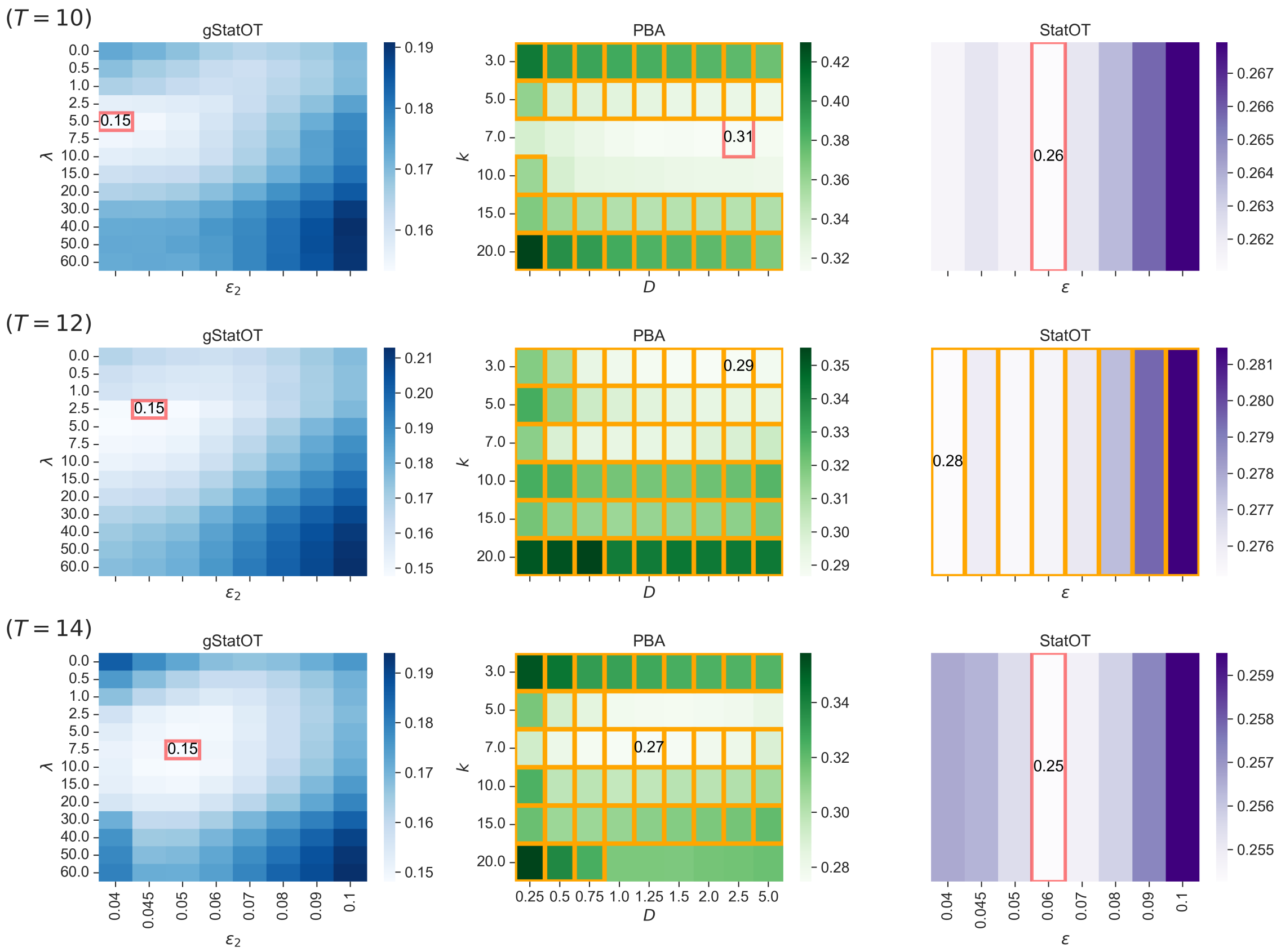

Figure SI.20: *Continuation of Figure SI.19.*

SI.8 Usage of LLMs

Large Language models were utilized to assist in writing improved code and documentation at https://github.com/ColeBoyle/global-StationaryOT.

References

[S1] Hugo Lavenant et al. "Toward a mathematical theory of trajectory inference". In: *The Annals of Applied Probability* 34.1A (2024), pp. 428–500.

[S2] Stephen P Boyd and Lieven Vandenberghe. *Convex optimization*. Cambridge university press, 2004.

[S3] Jonathan M Borwein and Qiji J Zhu. *Techniques of variational analysis*. Springer, 2005.

[S4] Gabriel Peyré, Marco Cuturi, et al. "Computational optimal transport: With applications to data science". In: *Foundations and Trends® in Machine Learning* 11.5-6 (2019), pp. 355–607.

[S5] Zhi-Quan Luo and Paul Tseng. "On the convergence of the coordinate descent method for convex differentiable minimization". In: *Journal of Optimization Theory and Applications* 72.1 (1992), pp. 7–35.

[S6] Stephen Wright, Jorge Nocedal, et al. "Numerical optimization". In: *Springer Science* 35.67-68 (1999), p. 179.

[S7] James Bradbury et al. *JAX: composable transformations of Python+NumPy programs*. Version 0.7.1. 2025. URL: http://github.com/jax-ml/jax.

[S8] DeepMind et al. *The DeepMind JAX Ecosystem*. 2020. URL: http://github.com/google-deepmind.

[S9] Caleb Weinreb et al. "Fundamental limits on dynamic inference from single-cell snapshots". In: *Proceedings of the National Academy of Sciences* 115.10 (2018), E2467–E2476.

[S10] Stephen Zhang et al. "Optimal transport analysis reveals trajectories in steady-state systems". In: *PLoS computational biology* 17.12 (2021), e1009466.

[S11] Philipp Weiler et al. "CellRank 2: unified fate mapping in multiview single-cell data". In: *Nature Methods* 21.7 (2024), pp. 1196–1205.

[S12] Marco Cuturi et al. "Optimal transport tools (ott): A jax toolbox for all things wasserstein". In: *arXiv preprint arXiv:2201.12324* (2022).

[S13] Aditya Pratapa et al. "Benchmarking Algorithms for Gene Regulatory Network Inference from Single-Cell Transcriptomic Data". In: *Nature Methods* 17.2 (2020), pp. 147–154. ISSN: 1548-7091, 1548-7105. DOI: 10.1038/s41592-019-0690-6.

[S14] Hojun Li et al. "The dynamics of hematopoiesis over the human lifespan". In: *Nature methods* 22.2 (2025), pp. 422–434.

[S15] Yuhan Hao et al. "Dictionary learning for integrative, multimodal and scalable single-cell analysis". In: *Nature Biotechnology* (2023). DOI: 10.1038/s41587-023-01767-y. URL: https://doi.org/10.1038/s41587-023-01767-y.

[S16] J. D. Hunter. "Matplotlib: A 2D graphics environment". In: *Computing in Science & Engineering* 9.3 (2007), pp. 90–95. DOI: 10.1109/MCSE.2007.55.

[S17] F Alexander Wolf, Philipp Angerer, and Fabian J Theis. "SCANPY: large-scale single-cell gene expression data analysis". In: *Genome biology* 19.1 (2018), p. 15.

[S18] Kazuaki Kishida. *Property of average precision and its generalization: An examination of evaluation indicator for information retrieval experiments.* National Institute of Informatics Tokyo, Japan, 2005.

[S19] Kevin P Murphy. *Probabilistic machine learning: an introduction.* MIT press, 2022.
